# Ultrasound-driven wireless piezoelectric hydrogel synergizes with cotransplantation of NSCs–hUCMSCs for structural and functional recovery in spinal cord injury

**DOI:** 10.1016/j.mtbio.2025.101805

**Published:** 2025-04-26

**Authors:** Hao Zhong, Mi Zhou, Junrui Guo, Danyang Chen, Cong Xing, Song Liu, Hongjiang Yang, Hongpeng Ma, Qi Zhang, Jianhai Yang, Shiqing Feng, Guangzhi Ning

**Affiliations:** a International Science and Technology Cooperation Base of Spinal Cord Injury, Tianjin Key Laboratory of Spine and Spinal Cord Injury, Department of Orthopedics, Tianjin Medical University General Hospital, 154 Anshan Road, Heping District, Tianjin, 300052, China; b School of Materials Science and Engineering Tianjin Key Laboratory of Composite and Functional Materials, Tianjin University, Tianjin, 300350, China; c Department of Orthopedics, Qilu Hospital of Shandong University, Shandong University Centre for Orthopedics, Advanced Medical Research Institute, Shandong University, Jinan, Shandong Province, China; d Orthopedic Research Center of Shandong University & Advanced Medical Research Institute, Cheeloo College of Medicine, Shandong University, Jinan, Shandong Province, China

**Keywords:** Spinal cord injury, Piezoelectric nanogenerator, Wireless electrical stimulation, Ultrasound

## Abstract

Spinal cord injury (SCI) is a devastating condition of the central nervous system, characterized by disrupted regulation of the immune microenvironment and the loss of electrical signaling, which poses significant challenges to repair. Neural stem cells (NSCs) have the potential to promote functional recovery after SCI; however, their therapeutic potential is limited by poor survival, restricted proliferation, and suboptimal differentiation. Human umbilical cord-derived mesenchymal stem cells (hUCMSCs) possess powerful paracrine and immunomodulatory properties, providing a supportive niche that improves the engraftment and function of NSCs. Recently, piezoelectric materials have attracted increasing attention for their ability to convert mechanical energy into electrical signals, thus providing a noninvasive, wireless alternative to traditional electrode-based therapies for neural regeneration. In this study, we investigated the synergistic effects of NSCs and hUCMSCs, focusing on how hUCMSCs direct NSC differentiation and the mechanisms underlying this action. We also introduce an ultrasound-driven wireless piezoelectric hydrogel, which generates electrical signals through the piezoelectric effect. In vitro, wireless electrical stimulation activated primary cortical neurons, stimulated axonal growth, and promoted neuronal plasticity through the Piezo1 channel and downstream CREB/CAMKII signaling pathways. In a rat SCI model, wireless piezoelectric hydrogel synergized with cotransplanting NSCs–hUCMSCs and modulated the immune microenvironment during the acute phase, thereby restructuring scar cavities during the chronic phase, suppressing scar formation, accelerating neurogenesis, and facilitating axonal regeneration. These results emphasize the potential of synergizing stem cell therapies with wireless piezoelectric stimulation as a promising strategy for SCI repair, providing novel insights into the clinical translation of regenerative treatments.

## Hightlights

• Developed a wireless-powered piezoelectric composite hydrogel for spinal cord injury.
• The hUCMSCs could enhances NSCs proliferation, and directed differentiation.
• Wireless electrical stimulation activates primary cortical neurons, stimulates axonal growth, and promotes neuronal plasticity via the Piezo1 channel and downstream CREB/CAMKII signaling pathways.
• The WPC hydrogel with electrical stimulation reduces cystic cavities and supports axonal regeneration in SCI.

## Graphical abstract

**Figure:**
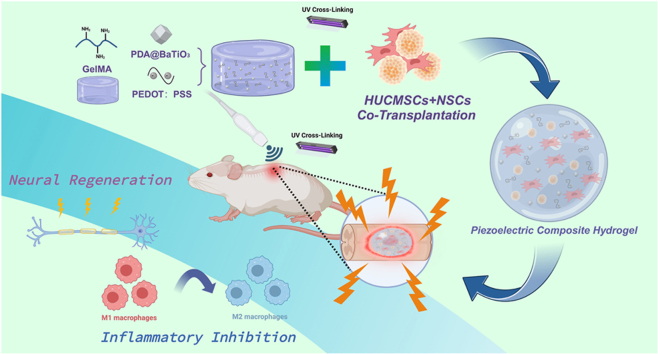

## 1 Introduction

Spinal cord injury (SCI) is a severe condition affecting the central nervous system, often causing sensory, motor, and autonomic dysfunction below the level of injury. This condition imposes substantial physical, emotional, and financial burdens on patients, their families, and society. The global incidence of SCI is estimated between 11 and 16 cases per 100,000 people annually, with falls and traffic accidents being the primary causes [1]. Currently, no universally effective method is available for successfully repairing SCIs, making it a major challenge in the field of neurology.

The pathological course of SCI is highly complex and generally divided into primary and secondary injuries. Primary injuries refer primarily to the compression or transection of the spinal cord caused by the initial mechanical trauma [2]. In contrast, secondary injuries encompass a complex array of pathological processes. In actue phase (<48h),tissue edema, activation of inflammatory cascades, and release of proinflammatory factors. The poor inflammatory microenvironment in injured areas hinders the full recovery from SCI, thereby complicating the healing process. In the subacute phase (2–14 days post-injury), continuous neuronal cell death occurs due to ischemia, inflammation, and excitotoxicity. The wave of microglial activation emerged, during which microglia clear cellular and myelin debris while releasing a large amount of reactive oxygen species, exacerbating inflammation and cell death. Subsequently, the injury gradually progresses into the chronic phase, characterized primarily by apoptosis and necrosis, axonal degeneration, axonal remyelination, axonal remodeling. Simultaneously, fluid-filled cystic cavitie expand over time, further damaging the spinal cord by obstructing axonal regeneration and disrupting electrical signal transmission, thereby exacerbating the severity of the injury [3].

Therapies based on stem cell transplantation have become an ideal candidate for the treatment of SCI. Neural stem cells (NSCs) are vital multipotent cells in the neural lineage, essential for neurogenesis and SCI repair strategies [4]. Nevertheless, preclinical research indicates that the reparative properties of neural progenitor cells are significantly affected by the imbalanced microenvironment following injury. Mesenchymal stem cells (MSCs) are a class of multipotent stem cells that can be obtained from a range of tissues, including but not limited to human umbilical cord blood, bone marrow, and adipose tissue. MSCs possess multidirectional differentiation and self-renewal capabilities, enabling them to differentiate into various terminal cell types such as adipocytes, chondrocytes, and neurons. Recent studies have shown that MSCs exhibit favorable immunomodulatory, neuroprotective, and neuroregenerative properties [[5], [6], [7], [8]]. Human umbilical cord-derived mesenchymal stem cells (hUCMSCs) possess several unique advantages compared with MSCs obtained from other sources, including low immunogenicity, low risk of infection, nontumorigenicity, and broad pluripotency [9]. NSCs transplantation faces challenges due to the lack of essential niche factors. hUCMSCs are recognized for their supportive role in regenerative medicine, primarily due to their paracrine signaling effects and immunomodulatory capabilities. These properties help create a comprehensive niche environment containing essential growth, nutritional, and regulatory factors for NSCs. Cotransplantation of NSCs and hUCMSCs forms a complex, multifactorial niche that has the potential to improve the therapeutic efficiency of NSCs.

The reshaping of bioelectrical signals plays a vital role in the functional recovery of axons after SCI [10,11]. The piezoelectric effect is a phenomenon where dielectric materials with asymmetric centers generate spontaneous polarization in response to external stress, resulting in the production of electrical signals on the material's surface [12]. BaTiO_3_ nanoparticles (BT NPs) are among the first piezoelectric materials to be investigated [13]. BaTiO_3_ can polarize and emit electrical signals under ultrasound, exhibiting excellent piezoelectric properties. Consequently, they can provide effective electrical stimulation without the need for implanted electrodes or external power sources [[14], [15], [16]]. Nevertheless, the incompatibility of BT NPs with neural tissue—structurally, mechanically, and chemically—limits their application in the repair of nerve injuries [17]. Gelatin methacryloyl (GelMA) hydrogels [18] are crosslinked functional polymer materials with high water content and can transmit mechanical, chemical, and electrical signals, exhibiting good biocompatibility. Due to the soft and hydrated nature, which resembles neural tissue, GelMA hydrogels possess mechanical properties similar to those of the neural tissue. GelMA could fill cavity spaces created by injuries and promote the regeneration of neural tissue [[19], [20], [21]]. Poly(3,4-ethylenedioxythio-phene): poly (styrenesulfonate) (PEDOT:PSS) shown to be the most promising conductive polymers for biomedical applications because of its excellent chemical stability, electrical properties, and biocompatibility [22].The incorporation of PEDOT:PSS to the GelMA matrix can significantly improve the electrical conductivity of the hydrogel. Under the noninvasive driving of ultrasound, BaTiO_3_ based conductive hydrogels can generate piezoelectric signals, providing in situ electrical stimulation at the center of SCIs, thus serving as a bridge for axonal regeneration.

**Fig. 1:**
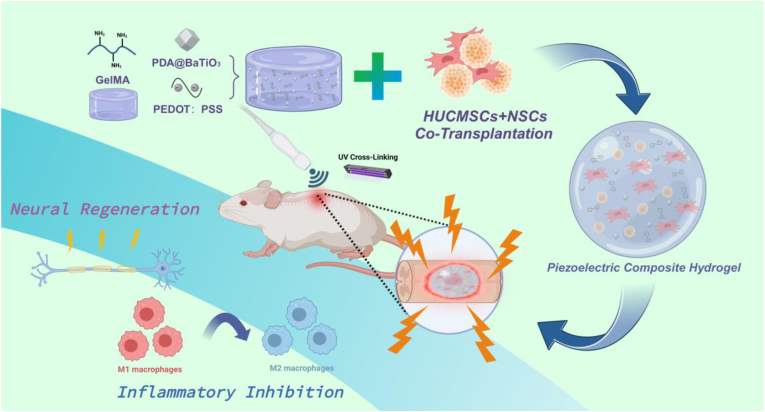
Schematic of the WPC hydrogel for recovery after SCI. The WPC hydrogel is activated by an external US to generate electrical cues. Created with BioRender.com.

In this study, we developed an ultrasound-driven, wireless-powered piezoelectric composite (WPC) hydrogel composed of polydopamine (PDA)-modified BaTiO_3_ nanoparticles (PDA@BT NPs), biocompatible conductive materials PEDOT:PSS, and GelMA hydrogel, which is loaded with NSCs and hUCMSCs (Fig. 1). During the subacute stage, the WPC hydrogel, in combination with wireless electrical stimulation (WES), promoted the switch of macrophage polarity from the proinflammatory M1 phenotype to the anti-inflammatory M2 phenotype. As the injury progressed to the chronic stage, this comprehensive strategy reduced mechanical stiffness of the scar tissue, restructured and alleviated cystic cavities, and resulted in improved tissue repair and locomotor function recovery in a clinically relevant contusive SCI model in rats. These findings suggest that the piezoelectric hydrogel, driven by ultrasound, synergizes with the cotransplantation of NSCs–hUCMSCs and provides a promising therapeutic approach for minimizing undesired scarring, thereby improving tissue repair and functional recovery after SCI.

## 2 Materials and methods

### 2.1 Synthesis and characterization of PDA@BT NPs

**Fig. 2:**
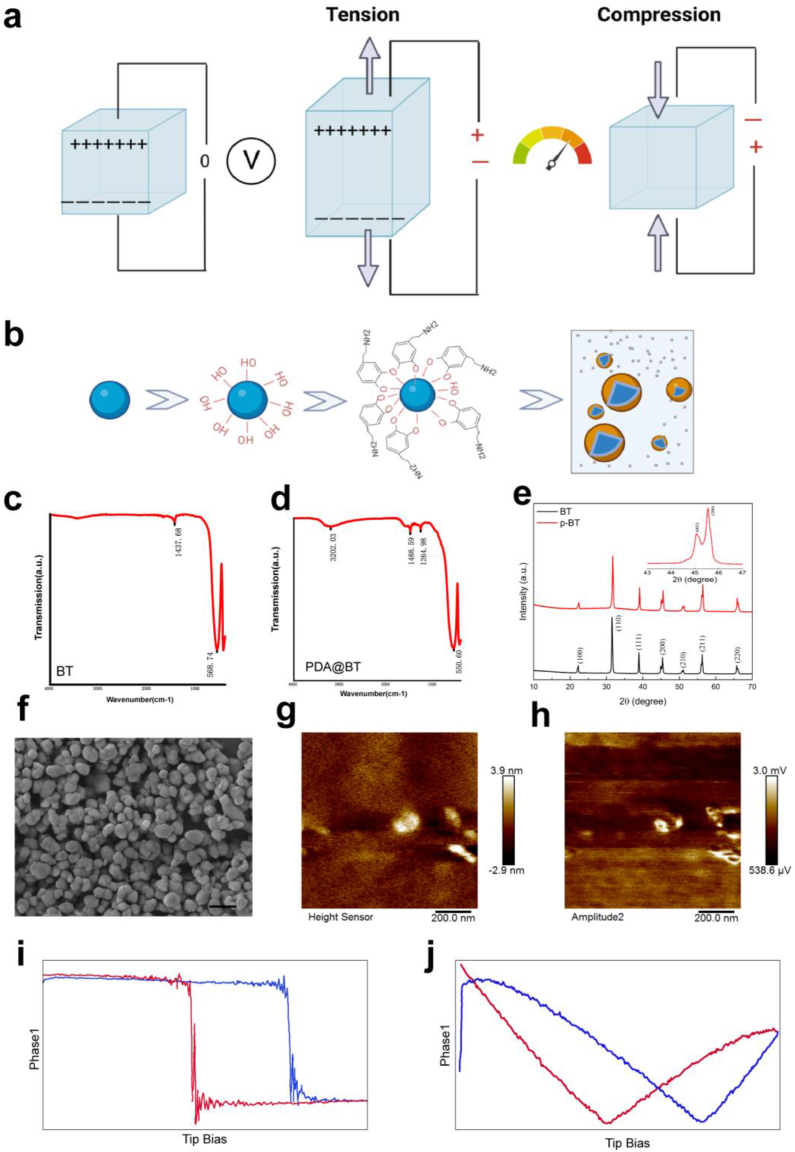
Characterization and piezoelectric properties of PDA@BT NPs. a) Schematic of piezoelectric signal generation. b) Schematic of the preparation of PDA@BT NPs. c, d) FT-IR spectra of BT NPs and PDA@BT NPs. e) XRD curves of BT and PDA@BT, respectively. f) TEM images of PDA@BT NPs. Scale bar = 500 nm. g, h) Images of topography g) and amplitude h) of PDA@BT NPs observed via PFM. i, j) Piezoresponse phase curves i) and amplitude–voltage j) PDA@BT NPs.

First, 1.21 g of Tris(hydroxymethyl)methyl aminomethane was dissolved in 800 ml of deionized water. The pH was adjusted to 8.5 using HCl solution. Then, 200 ml of DI water was added, and the pH was adjusted to 8.5 using HCl solution; this preparation is the Tris–HCl buffer solution. Next, 1.5 g of BT NPs (HBT-020, 200 nm, Shandong Sinocera, 200 nm) was uniformly dispersed in 200 ml of DI water to obtain the BaTiO_3_ dispersion solution. Subsequently, the BaTiO_3_ dispersion was mixed with 0.5 g of dopamine hydrochloride into 750 ml of the Tris–HCl buffer solution and stirred for 48 h at 25 °C, after which the mixture was centrifuged at 5000 rpm for 5 min. Then, the resulting product was washed five times with anhydrous ethanol. Finally, the sample was dried in a drying oven (80 °C) for 24 h to obtain PDA@BT NPs, as illustrated in Fig. 2b. Piezoresponse force microscopy images and amplitude-butterfly loops of PDA@BT NPs were obtained using an atomic force microscope (Dimension Icon, Bruker). Measurements were conducted in the PFM mode using a conductive probe and operating under contact mode conditions.

### 2.2 Characterization of piezoelectric hydrogel

The morphology and elemental composition of the piezoelectric hydrogels were examined by scanning electron microscope (SEM) coupled with energy-dispersive X-ray (EDX) mapping (Regulus 8100, HITACHI). The functional group changes in the hydrogels were analyzed using X-ray diffraction (D8, Bruker) and Fourier transform infrared spectroscopy (Nicolet iS10, Thermofisher). X-ray photoelectron spectroscopy (XPS) measurements were performed using an Escalab 250Xi (Thermofisher). To determine the piezoelectric properties of the developed hydrogels, we fabricated piezoelectric nanogenerators by integrating these hydrogels into the device architecture. After preparing the hydrogel, aluminum foil electrodes were attached to both sides of the hydrogel to fabricate a piezoelectric nanogenerator, which was then encapsulated with a waterproof layer of polydimethylsiloxane (PDMS). The voltage outputs under ultrasound stimulation were recorded using Data Acquisition Board (USB-6366, NI).

### 2.3 Rheological analysis

Rheological characterization of the WPC hydrogels was conducted using a rheometer (MCR302, Anton Paar, Austria). All tests were performed at 25 °C, and all experimental procedures were conducted at a constant temperature of 25 °C. The frequency sweep analysis was performed by systematically varying the frequency range from 10^−1^–10^2^ Hz. For the strain amplitude sweep tests, shear strain was incrementally increased from 10 % to 10,000 %. Moreover, alternating strain scans were conducted with the strain oscillating between 5 % and 50 %, maintained at each level for a duration of 60 s.

### 2.4 Cell culture

SH-SY5Y cells were cultured in high-glucose DMEM (Gibco) containing 10 % FBS. The hUCMSCs used in this study were provided by Changhe Biotechnology Inc. (Tianjin, China). The hUCMSCs were cultured in MEM (Thermo) supplemented with 5 % FBS. For the identification of hUCMSCs, the expression levels of the MSC surface markers CD14, CD90, CD105, and CD45 were analyzed by flow cytometry. Primary NSCs were extracted from the hippocampus region of the brain derived from wild-type Wistar rat (E14) embryos. Then, the NSCs were cultured in DMEM/F12 (1:1) (Gibico) supplemented with 2 % B-27 Supplement minus vitamin A (Gibico), 1 % GlutaMax (Gibico), 20 ng/mL bFGF (PeproTech), and 20 ng/mL EGF (R&D) for proliferation, with half of the medium being changed every other day until the neurospheres reached a size of 200 μm for the next step of the experiment. The differentiation culture medium was prepared by removing bFGF and EGF from the proliferation culture medium, and it was supplemented with 1 % FBS to facilitate the differentiation process. The NSCs and hUCMSCs were cocultured in a transwell system (0.4 μm, LABSELECT).

### 2.5 In vitro biocompatibility experiments

To evaluate the in vitro cytotoxicity of PDA@BT NPs, hUCMSCs and NSCs were plated in 96-well plates at an initial density of 1 × 10^4^ cells/well. After a 24-h incubation period, the cells were rinsed with PBS. Subsequently, PDA@BT NPs at different concentrations and PEDOT:PSS were added into each well, and the plates were returned to the incubator for 24 h at 37 °C. Cell viability was evaluated using the cell counting kit 8 (CCK-8) assay kit (Solarbio, China), strictly adhering to the manufacturer's protocol to analyze cellular proliferation. Furthermore, the Calcein/PI Cell Live/Dead Assay Kit (Beyotime, China) was used to differentiate live and dead cells. Observations were made under an inverted fluorescence microscope (Leica, Germany), where live cells exhibited green fluorescence, and dead cells exhibited red fluorescence.

### 2.6 SCI model establishment

All animal experiments were approved by the Animal Ethics Committee at Tianjin Medical University General Hospital (Approval No. IRB2023-DW-55). Adult female Wistar rats (weighing 180–220 g) provided by Vital River Laboratory Animal Technology (Beijing, China) were used in this study. When the rats were deeply anesthetized with 3 % isoflurane, the concentration of the anesthetic was subsequently reduced to 1.5 % for the maintenance of anesthesia. After performing T10 laminectomy to expose the spinal cord, spinal contusion injury was inflicted using the Impactor Model III (W.M. Keck Center for Collaborative Neuroscience, U.S.). 10-g weight was dropped from a height of 25 mm and immediately lifted after contacting the spinal cord, after which the muscle and skin were sutured in layer. Bladder care was provided twice daily until spontaneous voiding function recovered. After 7 days, the rats were subjected to anesthesia, and the dorsal part of the spinal cord was reexposed surgically. Before the resurgery, the NSCs and hUCMSCs for transplantation were centrifuged and stored temporarily at 4 °C. The contused area was reexposed, and 8 μl of PBS, or GelMA only, or wireless-powered piezoelectric (WP) hydrogel (composed of PDA@BT NPs, PEDOT:PSS, and GelMA hydrogel), or WPC hydrogel, was injected into the center of the injured spinal cord using a 10-μl Hamilton syringe under microscopy. Based on the varying injection compositions and subsequent treatment protocols, the rats were categorized into five distinct groups; the group that received PBS injection served as the SCI group, the group injected with the GelMA hydrogel was termed GelMA; the group injected with the WP hydrogel and received US stimulation was termed WP-US; the group injected with WP hydrogel-incorporated NSCs and hUCMSCs was termed WPC; and the group injected with wireless-powered piezoelectricWP hydrogel-incorporated NSCs and hUCMSCs and received US stimulation until the end of the experiment was termed WPC-US. The parameters of 20 min/day US stimulation were set to 1.0 MHz frequency, 2 W/cm^2^ intensity, and 20 % duty ratio. The 1.0 MHz frequency lasted till the end of the experiment.

### 2.7 Utilization of bioinformatics tools for the analysis of RNA-seq data

As part of the extraction and library construction process, we first measured the 260/280 ratio using a NanoDrop ND-1000 and a Bioanalyzer 2100. The average RIN value of RNA was 8. Thereafter, 1 μg of RNA was isolated using Dynabeads Oligo (dT)25–61005, followed by two rounds of purification. Poly(A)RNA was fragmented at 94 °C for 5–7 min before reverse transcription. Adapters with T-base overhangs were used to enable binding with DNA fragments containing A-tails by adding A-bases to each blunt end of the strands. The heat-labile UDG enzyme was used to label second-strand DNAs for PCR amplification after the ligation of single-index or dual-index adapters. Based on the manufacturer's instructions, paired-end sequencing (PE150) was performed on a NovaSeqTM6000 platform using the resulting cDNA library. Bioinformatics tools were used to process and interpret the RNA-seq data. The Fastp software was used to remove reads with adapter contamination and low-quality bases. Following assembler StringTie, GffCompare was used to reconstruct a transcriptome using all the mapped reads, and mRNA expression levels were calculated using gene counts. We then identified differentially expressed mRNAs using DeSeq2, using twofold change thresholds >2 and < 0.05, as well as a parametric F-test comparing nested linear models with a cutoff of <0.05. The sequencing work was commissioned by LC-Bio Technology CO., Ltd., Hangzhou, China.

### 2.8 Gene ontology (GO) annotation and pathway enrichment analysis

GO annotation of DEGs was performed using the clusterProfiler package. The complete annotation analysis involves BP, cellular composition, and MF. We applied “org. Rn.eg.db” to convert gene symbols into entrezIDs. Kyoto Encyclopedia of Genes and Genomes (KEGG) enrichment analysis was used, and the results were visualized using the ggplot2 package. Gene set enrichment analysis (GSEA) analysis was performed using the **clusterProfiler** R package, and the gene set data were sourced from the **MSigDB** database.

### 2.9 Construction of protein–protein interaction (PPI) networks and module analysis

To explore the PPI, differentially expressed genes (DEGs) were submitted to the STRING database (http://string-db.org), a free and accessible database for screening and integrating data [23]. The sample type was “Rattus norvegicus.” The minimum interaction score of ≥0.4 was determined, and the disconnected nodes were hidden in constructing the PPI network. The Cytoscape software (version 3.10.3) was used for visualization [24]. Subsequently, the molecular complex detection plugin exploring the significant modules of the required network was used to identify core clusters with the criterion of node score cutoff = 0.2, degree cutoff = 2, k-score = 2, and max depth = 100. We depict the top four modules in this study.

### 2.10 Behavioral and footprint assessment

Locomotor function recovery after SCI was evaluated using the (Basso–Beattie– Bresnahan) BBB scores. Rats from each experimental group underwent behavioral testing at 1, 2, 3, 4, 5, 6, and 7 weeks postinjury. During the assessment, all animals were placed in an open field measuring 150 cm × 150 cm and observed for a minimum of 3 min by two independent examiners who were blinded to the animals’ group assignments to ensure unbiased evaluation of locomotor function. To further evaluate locomotor function recovery in rats in terms of gait, the CatWalk XT (the Netherlands) system was used to quantify footprints and gait parameters. Louisville Swim Scale (LSS) [25] scores was obtained on day 56 to evaluate hindlimb unction.

### 2.11 Immunofluorescence (IF) staining

The proliferation of NSCs was examined by the incorporation of EdU (Beyotime, China) for 4 h. For in vitro experiments, cultured cells were fixed with 4 % PFA for 15 min, followed by permeabilization with 0.25 % Triton X-100 and blocking with 5 % bovine serum albumin (BSA) for 1 h. Cells were then incubated overnight at 4 °C with the following primary antibodies: Tuj-1 (1:500, Abcam, USA) and GFAP (1:500, Cell Signaling Technology, USA),. Then, the cells were incubated with for 1 h with the following secondary antibodies: goat anti-rabbit 555 (1:500, Abcam, USA) and goat anti-mouse Alexa Fluor 488 (1:500, Abcam, USA). The cell nuclei were stained with DAPI for 15 min. Frozen sections were fixed with a 4 % PFA solution, followed by permeabilization with 0.25 % Triton X-100 and blocking with 5 % BSA, both performed at room temperature. The sections were incubated overnight at 4 °C with a panel of the following primary antibodies: anti-CD68 (1:200, Abcam, USA), anti-Arg1 (1:500, Abcam, USA), anti-iNOS (1:500, Invitrogen, USA), anti-GFAP (1:500, Abcam, USA), and anti-NF200 (1:200, Servicebio, USA). The next day, the frozen sections were incubated for 1 h with the following secondary antibodies: donkey anti-rabbit 647 (1:200, Abcam, USA), donkey anti-goat 555 (1:200, Beyotime, China), goat anti-rabbit Alexa Fluor 488 (1:500, Beyotime, China), and goat anti-mouse Alexa cy3 (1:200, Beyotime, China). Images were obtained using a confocal laser scanning microscope (Nikon, Japan).

### 2.12 Statistical analysis

Results were expressed as mean ± SD (standard deviation). H&E staining, immunostaining, and other imaging analyses were conducted using Fiji and GraphPad Prism software, version 8.0.2. Statistical evaluations were conducted using SPSS, version 23.0 (SPSS Inc., USA). One-way analysis of variance was used to reveal significant differences among groups, followed by Tukey's post hoc test for multiple comparisons. P < 0.05 was considered to denote statistical significance.

## 3 Results

### 3.1 Characterization and piezoelectric properties of PDA@BT NPs

Piezoelectric materials constitute a diverse subset of both inorganic and organic dielectric compounds characterized by their capacity to generate electrical polarization in response to mechanical stimulation (Fig. 2a) [26]. As the major transmitter of the central nervous system, PDA has been actively investigated as both nanoparticle and surface coating material during the recent 10 years [27]. BT NPs were first modified with PDA to improve their dispersibility and strengthen the interfacial interaction in the hydrogel matrix (Fig. 2b).

The FTIR spectra of BT and PDA@BT are depicted in Fig. 2c and d. A prominent peak was detected at 568 cm^−1^, attributed to the Ti-O vibration characteristic of BT. As anticipated, the FTIR spectrum of PDA@BT exhibited three new peaks compared with BT. The peak at 1264 cm^−1^ corresponded to the C-O stretching vibration of catechol groups, whereas the peak at 1488 cm^−1^ was attributed to the C-C stretching vibration of the benzene ring. Furthermore, hydroxylated BT NPs exhibited a broad peak at 3450 cm^−1^, primarily due to the stretching vibration of the O-H group.

BT NPs exist in multiple crystal phases. The cubic phase possesses a highly symmetric structure but does not exhibit piezoelectric properties. Therefore, in this study, we used the tetragonal phase BT NPs that exhibited excellent piezoelectric properties (Fig. 2f). The crystal structures of BT and PDA@BT were detected by XRD, as presented in Fig. 2e, which clearly shows that the double peak structure appeared at around 2θ of 45°, and the presence of two diffraction peaks suggests the presence of the tetragonal phase. The PDA coating did not compromise the crystal structures and physical properties of BT NPs; however, it prevented the current leakage and dramatic increase in dielectric loss [28]. The surface chemical composition and chemical bonding state of the modified PDA@BT were further analyzed by XPS, which showed that PDA binds to BT by forming the BT-O-C bond (Figure s1).

PFM is a mainstream technique for revealing and quantifying the piezoelectric properties of nanoscale particles [29]. PFM is a specialized atomic force microscope mode that uses alternating current voltage to measure piezoelectric coefficients. Fig. 2g and h depict the topography and amplitude of PDA@BT NPs observed by PFM. The topographical micrograph reveals an individual nanoparticle measuring approximately 200 nm, which is consistent with the expected size of the modified nanoparticles. The amplitude image reflects the local piezoelectric response, further confirming the successful modification of NPs. The local piezoresponse phase loop (Fig. 2i) reveals an approximately 180° phase shift caused by PDA@BT NPs, indicating reversible polarization between two antiparallel polarizations upon the application of an external electric field. Furthermore, the piezoresponse amplitude curve (Fig. 2j) exhibits an asymmetric butterfly-shaped pattern, along with a horizontal shift in the piezoresponse phase loops, because of the deformation of the material surface in response to voltage changes. These findings confirm the superior piezoelectric properties of PDA@BT NPs.

### 3.2 Synthesis and characterization of WPC hydrogel

The application of PDA@BT in neuroregeneration is hindered by chemical/mechanical incompatibility interfaces [17]. Good biosecurity is the basic requirement for biomaterial applications. Studies have confirmed that the GelMA/PEDOT:PSS conductive hydrogel PEDOT:PSS improves dorsal root ganglion and protects neurons [22,30]. The selected concentration of PEDOT:PSS of 1 mg/mL was based on the literature [31,32].

We first investigated the compatibility of the hydrogel leachate in vitro. Live-dead cell staining of SH-SY5Y cells cultured with hydrogel leachate for 24 h at 1, 3, and 7 days. Results showed that there was no effect on cell activity among groups with different concentrations of PDA@BTs (Figure s2a). hUCMSCs and NSCs were cultured on the hydrogel for 7 days in groups with different concentrations of PDA@BT. Live-dead cell staining indicated that there was no effect on cell activity after 7 days (Figure s2b). Moreover, the CCK-8 test was used to investigate the cytotoxicity and optimal concentration of PDA@BT on hUCMSCs and NSCs. As shown in Figure s2c, when the concentration of PDA@BT was >100 μg/ml, the viability of hUCMSCs was significantly affected, suggesting that PDA@BT at <100 μg/ml was not cytotoxic to hUCMSCs and NSCs. Therefore, we selected 100 μg/ml of PDA@BT as the optimal concentration for preparing the WPC hydrogel.

**Fig. 3:**
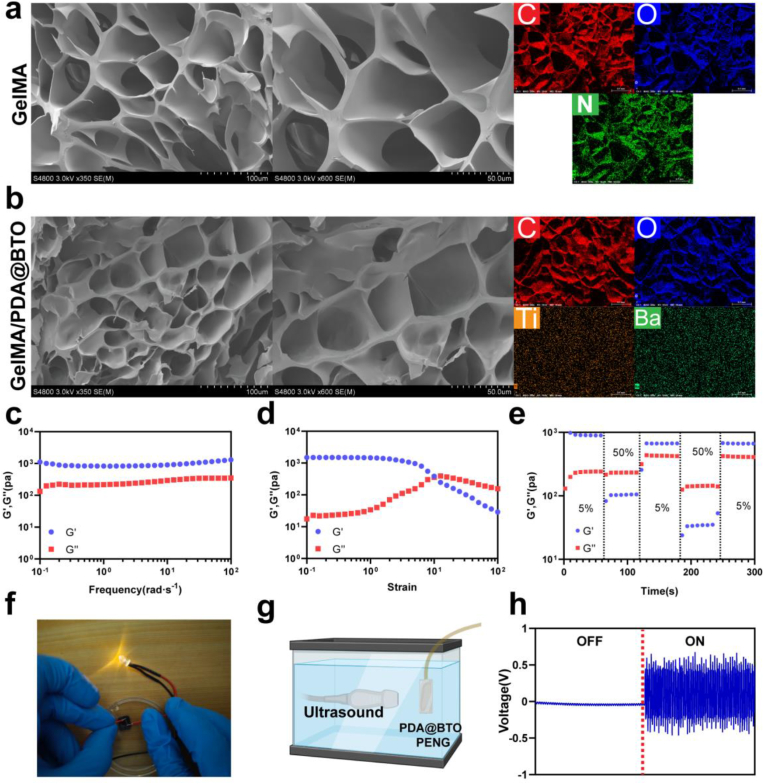
Characterization and piezoelectric properties of the PEDOT/PDA@BT hydrogel. a, b). SEM images and EDX surface-scan element distribution of GelMA a) and WPC hydrogel b). (Scale bar = 100 and 50 μm, respectively.) c-e) Rheological properties of WPC hydrogel. f) Images visually demonstrate the conductivity of the PEDOT/PDA@BT hydrogel. g) Schematic of the electrical output of piezoelectric nanogenerator (PENG) made of WP hydrogel under US activation. h) The open-circuit voltage generated by the piezoelectric nanogenerator under US activation.

Next, we examined the microstructure characterization of the hydrogel using SEM (Fig. 3a and b), which showed that GelMA and WPC hydrogel have a well-porous and honeycomb structure with relatively uniform pore size, which provided a suitable surface structure for cell adherent growth. Using EDX-mapping analyses, we demonstrated the successful incorporation of PDA@BT NPs into the hydrogel with uniform distribution.

Rheological frequency sweep curves of the WPC hydrogel indicated that the storage modulus (Gʹ) approached 1000 Pa and was significantly greater than the loss modulus (Gʺ) over an angular frequency range of 10^−1^–10^2^ Hz (Fig. 3c). According to previous research, for effective nerve repair, the mechanical properties of the hydrogel must closely mimic those of the spinal cord tissue, which should be within the range of 100–3000 Pa[33]. Therefore, the WPC hydrogel could provide good mechanical support for the repair of nerve cells and tissues. For further investigating the rheological properties, we examined the WPC hydrogel in the strain scan mode (Fig. 3d). In regions of low strain, the hydrogel exhibited consistent mechanical stability, with both the storage modulus (Gʹ) and loss modulus (Gʺ) remaining constant, indicating that the hydrogel network maintains its structural integrity and resistance to deformation. However, after reaching a critical strain of 1000 %, the Gʹ and Gʺ values experienced a sharp decline due to the collapse of the hydrogel network. After exposure to step-strain changes as depicted in Fig. 3e, the PDA@BT hydrogels demonstrated an immediate recovery to their original storage modulus (Gʹ) upon transitioning from a 50 % strain to a 5 % strain. These results suggests good stability of the WPC hydrogel. As shown in Fig. 3f, a segment of the WPC hydrogel was integrated into a closed electrical circuit connected to a light-emitting diode. Due to the high electroconductivity of the hydrogel, it was capable of sustaining a continuous flow of electric current through the circuit. To explore the piezoelectric performance of the WPC hydrogel, we fabricated a piezoelectric nanogenerator composed of the WPC hydrogel, Au electrodes, and PDMS encapsulation (Fig. 3g). US (2 W/cm^2^, 1 MHz) was applied to the nanogenerator made of the WPC hydrogel. As depicted in Fig. 3h, the peak-to-peak output voltage (Vpp) of the WPC hydrogel nanogenerator is 0.96 ± 0.13 V. These data confirm that the WPC hydrogel can effectively generate electrical signals when subjected to US stimulation, thereby facilitating wireless-powered piezoelectric ES.

### 3.3 hUCMSCs induce NSCs proliferation and neuronal differentiation

Before using the cells, we confirmed their identity by flow cytometry and Immunofluorescence. The hUCMSC preparations demonstrated high expression levels of the characteristic MSC markers CD90-positive (>99.70 %) and CD105-positive (>97.2 %) cells and negative surface markers of MSCs CD14 (2.66 %) and CD45 (12.6 %) (Fig. S3a).Identification of NSCs through cell morphology and the expression of nestin and SOX2 (Fig. S3b).

**Fig. 4:**
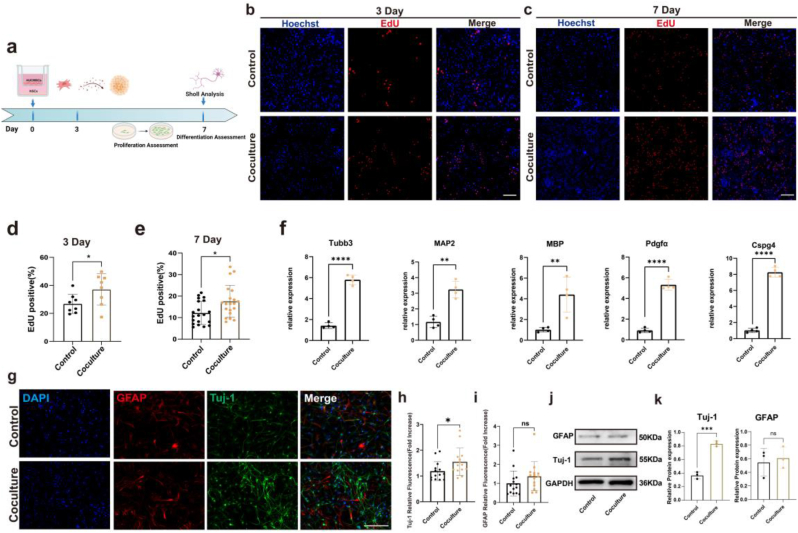
hUCMSCs induce NSCs proliferation and differentiation. a) Schematic of the evaluation timeline of coculture. Created with BioRender.com. b-e) Proliferation of NSCs determined using the EDU assay. Scale bars, 100 μm. f) RT-qPCR analysis of the expression of the neuron-specific genes Tuj-1, MAP2, MBP, PDGFα, and CSPG4 in NSCs cultured with hUCMSCs for 7 days. g) Representative images of Tuj-1/GFAP/nuclear immunostaining of NSCs. Scale bars, 100 μm. h, i) Quantification of the differentiation rates of NSCs. j) Representative western blotting images of Tuj-1 and GFAP of NSCs that received different treatments; GAPDH was used as the reference protein. k) Quantitative analysis of the expression level of Tuj-1 and GFAP. (ns indicates no significant, ∗p < 0.05, ∗∗p < 0.01, ∗∗∗p < 0.001, ∗∗∗∗p < 0.0001).

To explore the effects of hUCMSCs on NSCs, we used a transwell chamber to construct a coculture model, wherein NSCs were placed in the lower compartment of the transwell chamber, and hUCMSCs were seeded in the upper compartment. After coculturing with hUCMSCs for 3 and 7 days, the proliferation of NSCs was measured by EdU staining, which demonstrated that hUCMSCs could promote the proliferation of NSCs (Fig. 4b–e). After 7 days of coculture with hUCMSCs, NSCs were harvested for quantitative RT-PCR in which the following proteins expressed during differentiation were examined: βIII-tubulin, a specific marker for neural progenitor cells; MAP2, a marker of mature neurons; oligodendroglial markers MBP and Pdgfα; and the microglia marker CSPG4. The results of qRT-PCR showed that hUCMSCs promoted NSC neuronal differentiation (Fig. 4f). Moreover, we performed immunofluorescence staining to qualitatively evaluate neuronal differentiation (Fig. 4g). We observed that more Tuj-1+ cells appeared after coculture with hUCMSCs, suggesting that coculture could substantially improve the intrinsic neuronal differentiation of NSCs and induce NSCs to differentiate into neurons rather than astrocytes (Fig. 4h and i). We also conducted western blotting to measure the expression levels of proteins. The results of grayscale statistics revealed that the expression levels of Tuj-1 after coculture were 2-fold those in the CTL group. However, GFAP expression levels exhibited no significant changes.

### 3.4 Mechanism of coculture-induced directed differentiation of NSCs

**Fig. 5:**
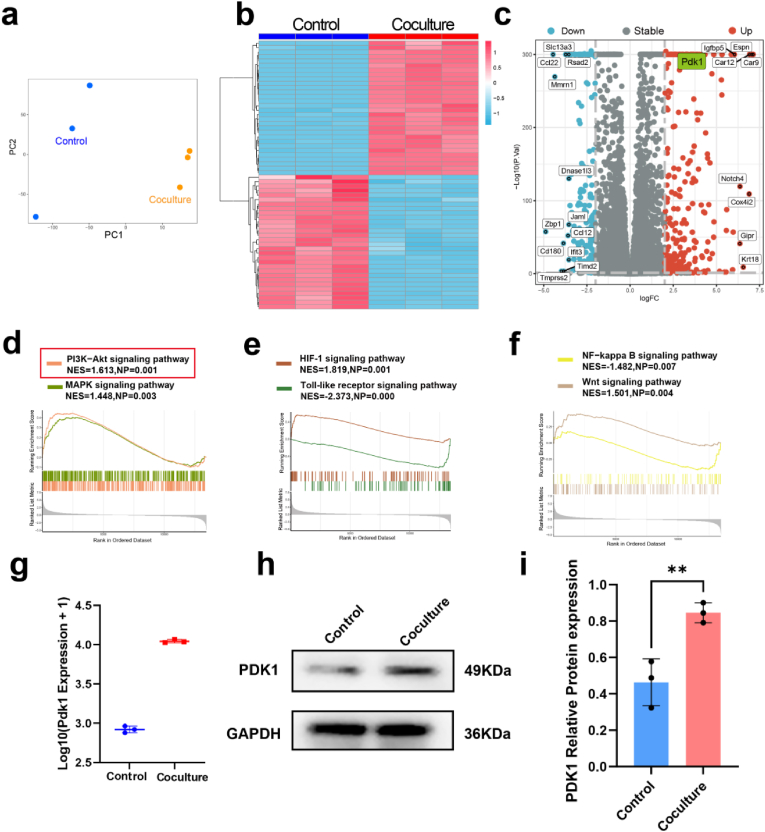
Identification of DEGs in the control and coculture groups. a) Principal component analysis (PCA) of transcriptome sequencing samples b) Heatmap of DEGs. c) Volcano plot of the identified DEGs. d-f) GSEA to show the enriched biological processes in cocultured NSCs. g) PDK1 expression in NSCs in gene level. h) Representative western blotting images of PDK1. i) Quantitative analysis of the expression level of PDK1. (∗∗p < 0.01).

To clarify the underlying mechanisms by which hUCMSCs promote the directed differentiation of NSCs into neurons, we performed transcriptome sequencing analysis. After a 7-day period of in vitro coculture, NSCs from the lower compartment were harvested for mRNA transcriptome sequencing. Reliability of the tested samples was evaluated using PCA (Fig. 5a), which revealed a high degree of similarity among the biological replicates of each sample, indicating that the sequencing data were relatively reliable and suitable for further analysis. Within the generated heatmap, we observed significant differences in the expression of DEGs between the coculture and control groups (Fig. 5b). These disparities probably disclose the molecular mechanisms underlying the interaction between two seed cells, providing novel insights into the impact of the coculture environment on the behavior of NSCs.

By applying a significance threshold of *P*-adj <0.05 and a fold change cutoff of |logFC| > 2, 463 DEGs were distinguished between the coculture and control groups. Volcano plots are used to gain insights into the global distribution of DEGs. These plots were constructed with log2(fold change) on the x-axis and -log10 (*P*-adj) on the y-axis, depicting all genes from the differential expression analysis. In these plots, red dots correspond to significantly upregulated genes, blue dots denote significantly downregulated genes, and gray dots represent genes with no significant change in expression levels (Fig. 5c). These results suggest that the coculture model with hUCMSCs significantly affected NSCs compared with the conventional in vitro culture model of normal NSCs.

Through GSEA (Fig. 5d–f), we identified significant enrichment of the PI3K-AKT pathway, with PDK1 emerging as a vital gene within this pathway. Furthermore, differential expression analysis using the DESeq2 package and western blotting confirmed that PDK1 exhibited significant differential expression (Fig. 5g–i), suggesting its potential as an important target. Supplementary Figure s4 shows the results of the enrichment analysis of genes related to the PI3K-AKT pathway, revealing five associated pathways that were also significantly enriched. These pathways are known to play major roles in cellular processes such as proliferation and differentiation.

**Fig. 6:**
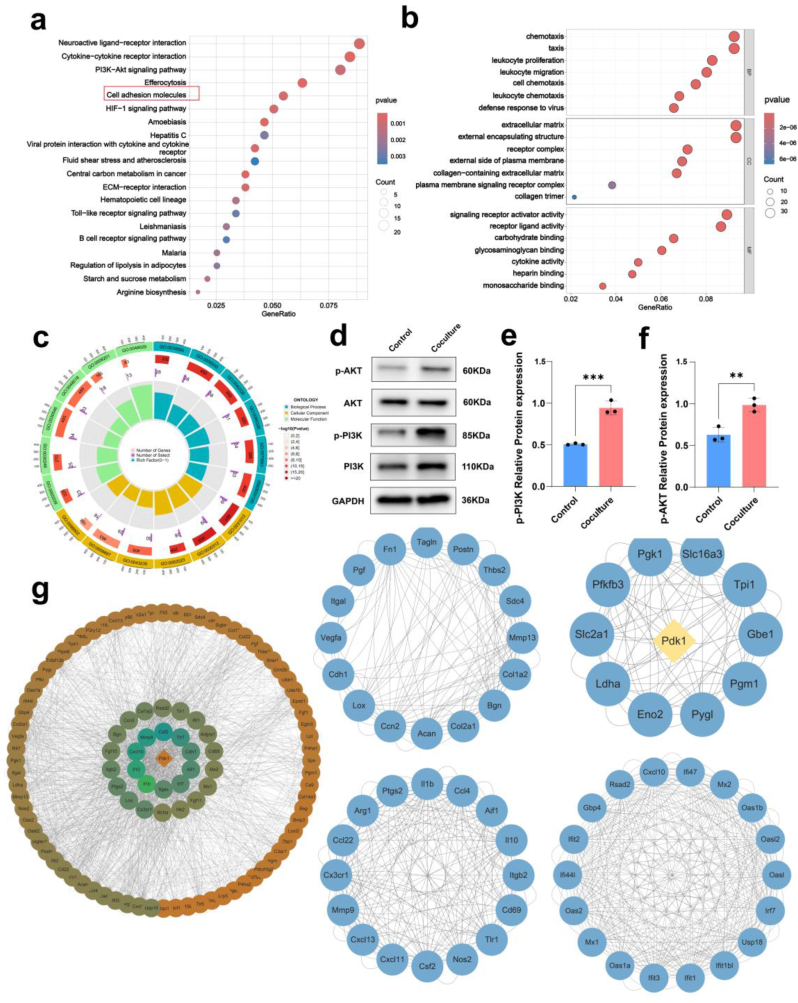
The mechanism by which hUCMSCs regulate the cell fate of NSCs. a) KEGG pathway enrichment analysis depicted the major enriched pathway among upregulated genes in Cocultured NSCs. b) Enrichment of GO biological process based on differentially expressed genes between the control and coculture groups. c) Pie chart showing the enriched GO terms. d-f) Western blot images showing the activation of PI3K/AKT pathway proteins in NSCs after coculture with hUCMSCs. g) Visualization of PPI protein interaction network analysis. (∗∗p < 0.01, ∗∗∗p < 0.001, ∗∗∗∗p < 0.0001).

The KEGG pathway enrichment analysis, supported by corresponding diagrams, indicated a significant enrichment of DEGs in several important pathways, including the PI3K-AKT pathway (Fig. 6a). GO functional enrichment analysis identified DEGs that were significantly enriched in the biological process (BP), cellular component (CC), and molecular function (MF) categories, providing insights into the classification of GO enrichment (Fig. 6b). The terms from BP, CC, and MF were sorted based on the number of DEGs they annotated, from the highest to the lowest. The top seven terms were selected for graphical representation. Regarding BP, the DEGs were associated with chemotaxis and taxis. At the CC level, the DEGs were enriched in the extracellular matrix (ECM) and plasma membrane. Regarding MF, the DEGs were predominantly involved in signaling receptor activator activity and receptor ligand activity. Fig. 6c shows the pie chart depicting the GO terms summarized per category according to their GO classification; the expansions of the abbreviated GO terms are provided in supporting Table 1. We next aimed to identify the key proteins involved in the observed processes by constructing PPI networks based on information from the STRING database regarding the differentially accumulated proteins in both the coculture and control groups. The resulting PPI network consisted of 340 nodes (proteins) connected by 742 edges (interactions), providing a visual depiction of the intricate interactions among these proteins. A module within a network is defined a set of nodes that are densely connected within subsets of the network but may not all directly interact with each other. To obtain further insight into the potential targets, we identified four modules in the PPI network. The central node, Pdk1, serves as a critical hub connecting multiple gene clusters, indicating its pivotal regulatory role in this network (Fig. 6g). Overall, the sequencing results demonstrated that the PI3K-Akt signaling pathway contained a large number of DEGs, suggesting it as the primary regulatory mechanism. The PI3K-Akt signaling pathway is a critical intracellular pathway that plays a vital role in regulating cell growth, proliferation, survival, and metabolism. Western blotting and statistical analysis revealed that the protein expression levels of p-PI3K/PI3K and p-Akt/Akt in cocultured NSCs were in excess of NSCs (Fig. 6d–f), showing that hUCMSCs induced the differentiation of NSCs into neurons potentially by activating the PDK1-driven PI3K-Akt signaling pathway.

### 3.5 WPC hydrogel WES activates Piezo1 channel regulating neuronal plasticity through the CaMKII/CREB pathway in neurons

**Fig. 7:**
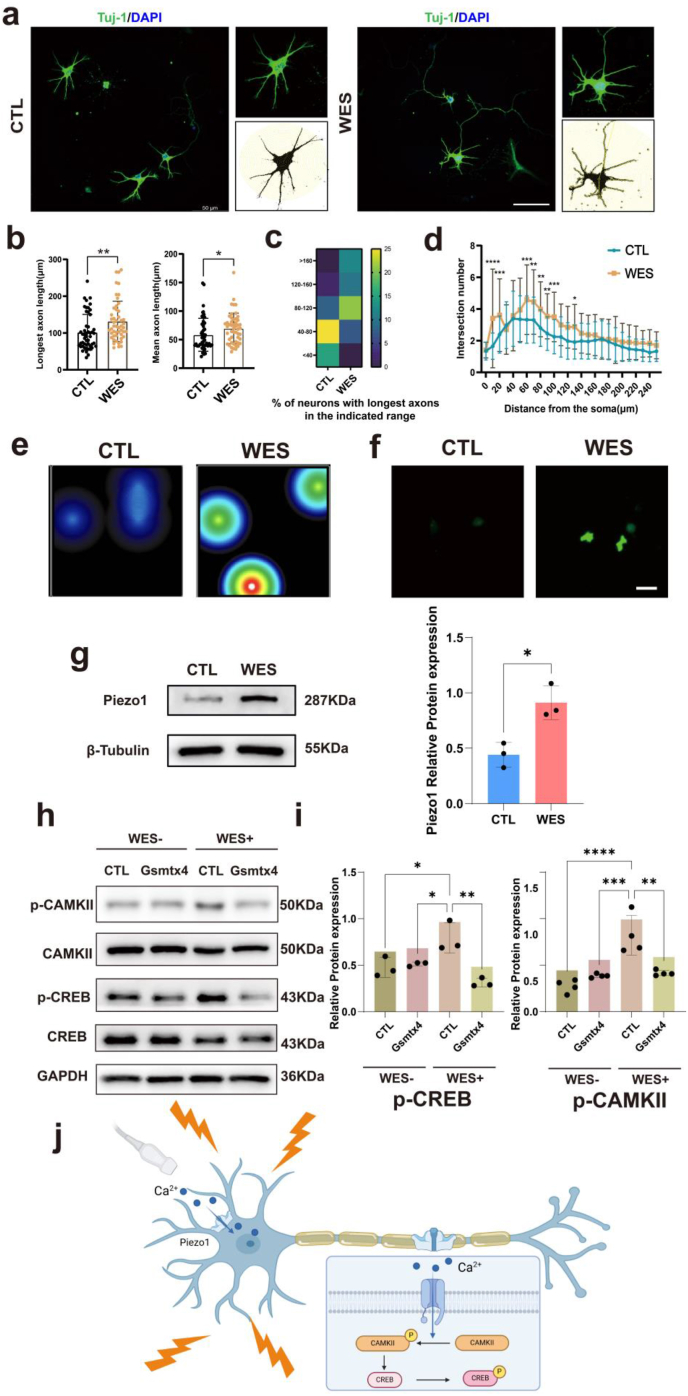
WPC hydrogel-facilitated wireless electrical stimulation(WES) activates Piezo1 channel regulating neuronal plasticity through CaMKII/CREB in neurons. a) Tuj-1 immunostaining of neurons cultured for 7 days. Scale bar = 50 μm. b-d) Sholl analysis of dendritic morphology in neurons. e) Heatmaps of spontaneous firing of neurons. f) Representative images of real-time calcium fluorescence image of neurons. Scale bar = 20 μm. g) Expression of Piezo1 in neurons. h, i) Representative western blot images and quantitative analysis of the expression level of p-CAMKII/CaMKII and p-CREB/CREB in neurons. j) Schematic of the mechanism of WES in regulating neuronal plasticity. Created with BioRender.com. (∗p < 0.05, ∗∗p < 0.01, ∗∗∗p < 0.001, ∗∗∗∗p < 0.0001).

Next, we investigated the effects of WES on primary cortical neurons to evaluate the feasibility of stimulating neurons with electrical signal. For neurons, the complexity of axons and dendrites was quantified using Sholl analysis (Fig. 7a). WES significantly promoted the longest axon length and mean axon length of neurons (Fig. 7b and c). Using Sholl analysis, we counted the number of intersections the dendrites made with the concentric spheres and found that the coculture increased the dendritic arbor complexity of neurons (Fig. 7d). Therefore, we concluded that WES could facilitate and improve neurite outgrowth in neurons. We next measured the electrical activity using microelectrode array (MEA). Neurons were seeded in 24-well MEA plates, and recordings were obtained 14 days after seeding. The heatmap revealed that WES increased neuronal activity (Fig. 7e).

The calcium signaling pathway is a crucial component of intracellular communication that regulates a diverse array of cellular processes. To better understand how WES affects neurons, we performed real-time Ca^2+^ imaging, wherein Fluo-4AM was conducted to detect the concentration of intracellular Ca^2+^. Results showed that WES resulted in an increase in Ca^2+^ influx into neurons (Fig. 7f). Moreover, as a mechanically sensitive ion channel that regulates Ca^2+^ influx, Piezo1 expression was significantly improved under WES treatment (Fig. 7g), suggesting that WES could promote Ca^2+^ influx by activating Piezo1 channel. We also explored in greater detail the signaling implications of Piezo1-mediated WES effects on neurons to clarify the possible downstream effects of Ca^2+^ influx through ultrasound-activated Piezo1 channels. To examine the effect of treatment on cell signaling downstream of the observed Ca^2+^ influx, we investigated the levels of the phosphorylated forms of Ca^2+^/calmodulin-dependent protein kinase type II (p-CaMKII) and the phosphorylated forms of the cAMP-response element binding protein (p-CREB). We determined the levels of these activated proteins by western blotting to examine whether WES could affect activation downstream of Ca^2+^ influx.

To determine whether the effects of WES on neurons depend on the activation of the Piezo1 channel, we used GsMTx4 to specifically inhibit Piezo1 activation. As shown by the western blotting results, the phosphorylation levels of CaMKII and CREB in WES-treated neurons increased compared with those in the WES(−) group. However, GsMTx4 at 1 μM significantly inhibited the WES-mediated p-CaMKII and p-CREB activation (Fig. 7h and i). Therefore, we determined that the WES-mediated Piezo1 channel activation in neurons could regulate neuronal plasticity and promote neuronal axon regeneration through CaMKII/CREB signaling (Fig. 7j).

### 3.6 WPC hydrogel WES inhibits inflammatory response and regulates macrophage/microglia polarization after SCI in vivo

**Fig. 8:**
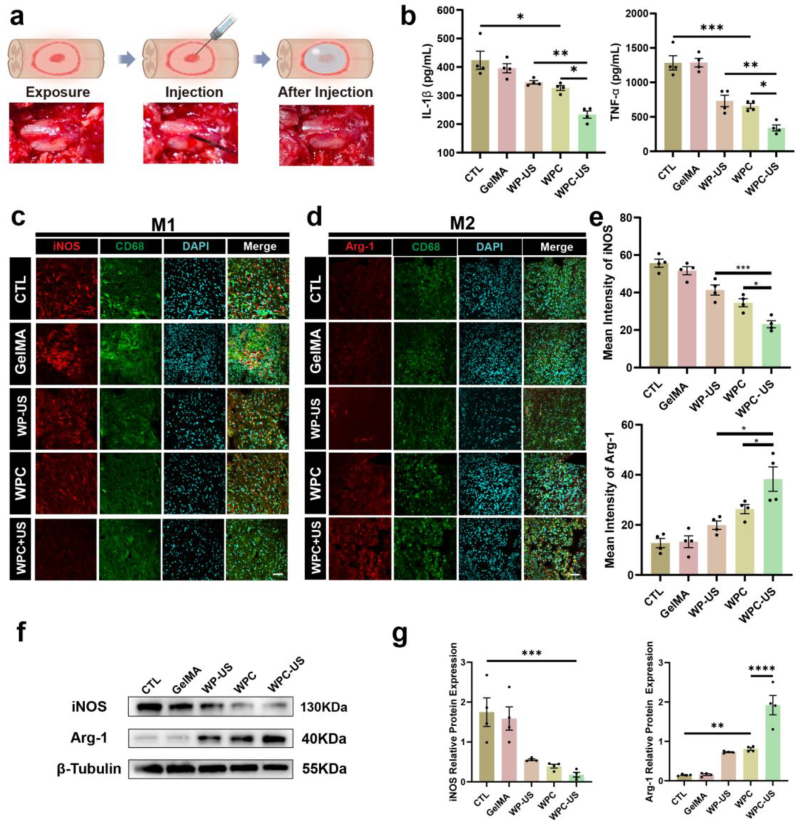
WPC hydrogel inhibits inflammation and regulates macrophage/microglia polarization in the acute phase after SCI. a) Illustration of the process of hydrogel injection. b) ELISA analysis of the concentrations of TNF-α and IL-1β proteins in spinal cord tissues. c) Immunofluorescence image of iNOS expression in macrophage/microglia. Scale bar = 100 μm. d) Immunofluorescence images of Arg-1 expression in macrophages/microglia. Scale bar = 100 μm. e) Quantitative analysis of the mean fluorescence intensity of iNOS and Arg-1 protein expression. f, g) Representative western blot images and quantitative analysis of the expression level of iNOS and Arg-1. (∗p < 0.05, ∗∗p < 0.01, ∗∗∗p < 0.001, ∗∗∗∗p < 0.0001).

The hydrogels were injected directly into the cystic cavities after a week to evaluate the therapeutic effect (Fig. 8a). During the subacute phase of SCI, to evaluate the capacity of the WPC hydrogel to alleviate the inflammatory microenvironment, spinal cord tissues were collected 3 days postinjection for further analysis. For verification of the anti-inflammatory effect, we quantitatively measured inflammatory cytokines by ELISA tests in each group. SCI showed significantly higher concentrations of TNF-α and IL-6 in spinal cord tissue. WPC-US group substantially reduced these inflammatory cytokines levels than other treatments (Fig. 8b). CD68 serves as a marker for identifying macrophages/microglia, and iNOS and Arg-1 are used to distinguish between the M1 and M2 polarization states of these immune cells, respectively. Immunofluorescence imaging revealed significantly downregulated expression of iNOS in both the WPC and WP-US groups compared with that in the SCI and GelMA groups. Remarkably, the WPC-US group exhibited the most pronounced reduction in iNOS expression. Compared with that in the SCI and GelMA groups, the expression of Arg-1 was upregulated in both the WPC and WP-US groups, with the most significant upregulation detected in the WPC-US group (Fig. 8c-e). Western blot analysis was further employed to validate the impact of treatments on the polarization of macrophages/microglia. The results revealed that WPC-US decreased the protein levels of M1 pro-inflammatory marker iNOS, while concurrently enhancing the expression of M2 anti-inflammatory marker Arg-1 (Fig. 8f and g). These findings demonstrated that WPC with WES could inhibits inflammatory response and regulates transformation of M1 macrophages/microglia into M2 macrophages/microglia, illustrating the potential of regulating the microenvironment of SCI.

### 3.7 WPC hydrogel with WES reduces scar formation, eliminates cystic cavities, and improves transduction function after contusive SCI

**Fig. 9:**
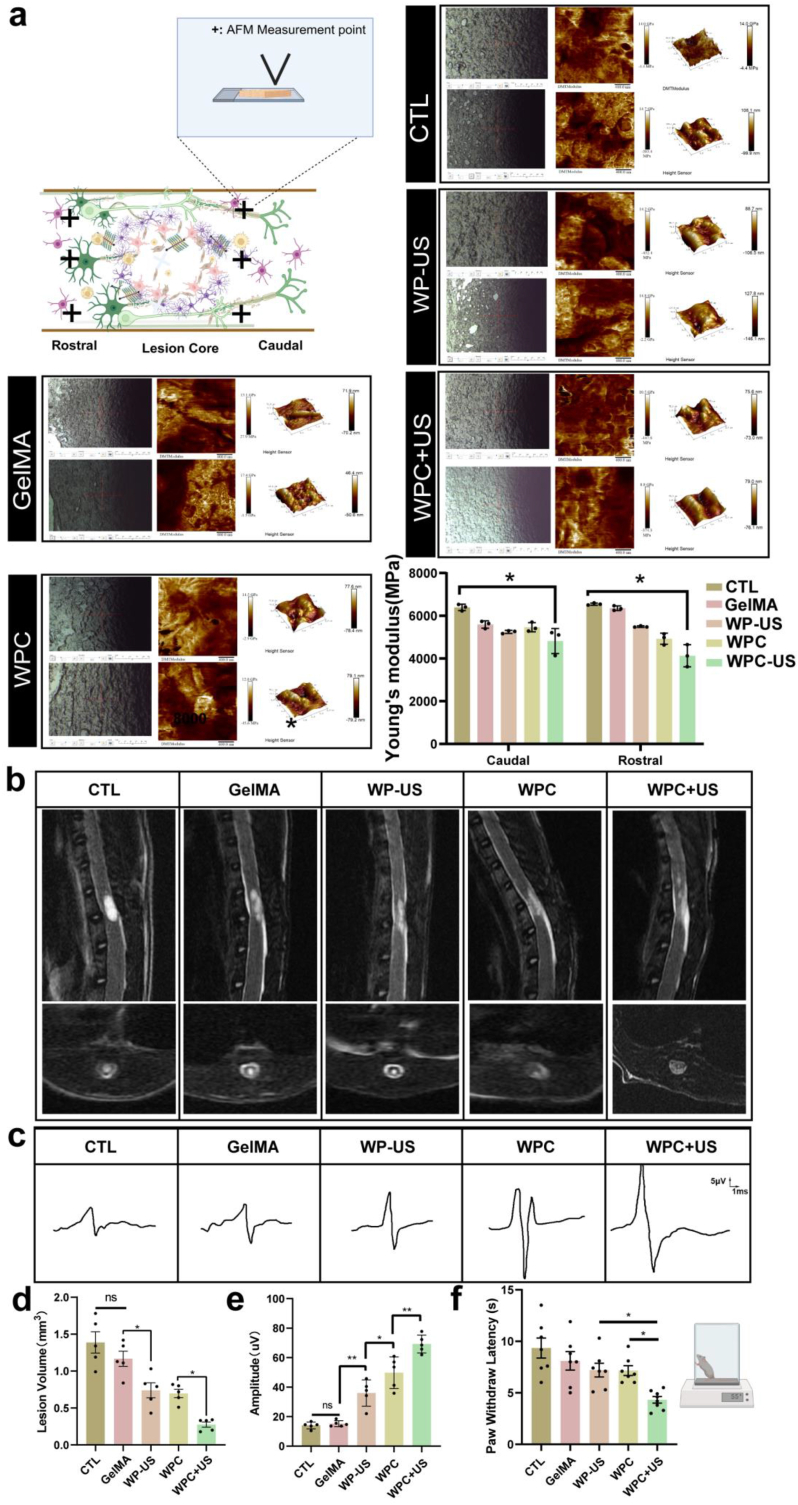
WPC hydrogel with wireless electrical stimulation reduces scar formation and lesion cavity. a) AFM stiffness characterization of scar tissue. b) Sagittal and axial T2-weighted images of rats in different groups. c) Representative electrophysiological images of Motor evoked potentials (MEP). d) Quantitative analysis of lesion cavity volume. e) Quantitative analysis MEP signals in each group (n = 5). f) Latency to withdrawal in the hot plate test. (ns indicates no significant, ∗p < 0.05, ∗∗p < 0.01).

The elevated stiffness of scar tissue inhibits axonal extension and restricts the migration of neural cells and other repair-associated cells essential for regeneration and functional recovery. To clarify the effects of the WPC composite hydrogel on scar stiffness, we used PFM to measure the changes in matrix stiffness among different treatment groups. Results showed that the WPC-US group exhibited the lowest modulus of scar tissue, demonstrating that WPC-US treatment effectively reduced the stiffness of ECM at the scar site. This reduction in matrix rigidity not only inhibits scar formation (Fig. 9a) but also generates a supportive microenvironment conducive to tissue regeneration. Such a microenvironment facilitates the regeneration of axons, enabling them to overcome the mechanical barrier posed by the scar tissue.

After confirming the reduction in scar stiffness, we further investigated the changes in the volume of cystic cavities by MRI to quantitatively examine the presence of cystic cavities at 8 weeks postinjury. Different lesion regions of the spinal cord in different groups were observed in sagittal and axial T2-weighted images. The volume of cystic cavity was calculated using the sagittal and axial images. As depicted in Fig. 9b–d, compared with that in the SCI and GelMA groups that showed severely damaged tissues and distinct cystic cavitation in the injured spinal cord, the relative cavity volume of the damaged spinal cord in the GelMA + Cell and WPC groups was significantly smaller. Moreover, WPC hydrogel with wireless ES resulted in an almost complete disappearance of cystic cavities.

We conducted electrophysiological measurements to evaluate the spinal nerve conduction function. The representative MEP peak almost disappeared in the SCI and GelMA groups. MEP significantly recovered, with amplitudes being higher in the GelMA + Cell and WPC groups than in the SCI and GelMA groups. In addition, electrophysiology experiments demonstrated a significantly higher amplitude of Motor evoked potentials signals in the group treated with the WPC hydrogel and WES than in the groups that received single treatments (Fig. 9c–e). In the hot plate experiment, rats were placed on a hot plate (55 °C ± 0.5 °C) to examine thermal sensitivity at 56 dpi (Fig. 9 f). Paw withdrawal latency is defined as the time since the foot touches the hot plate until the rat lifted or licked its hind paws. The SCI and GelMA groups showed a loss of temperature sensitivity. Significant deficits remained in the WP-US and WPC groups, whereas the latency of recognition of the hot plate further recovered in the WPC-US group.

### 3.8 WPC hydrogel with WES promotes locomotor function recovery after SCI

**Fig. 10:**
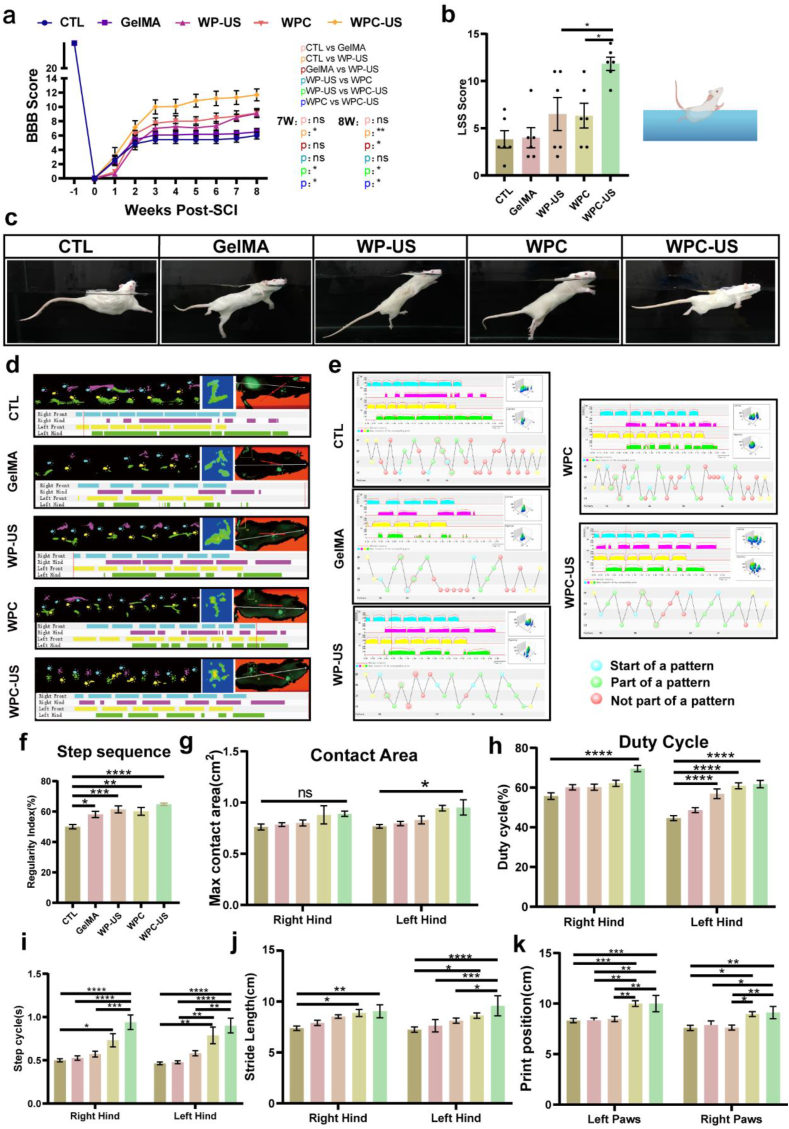
WPC hydrogel with WES promotes locomotor function recovery after SCI. a) BBB scores from preinjury to 8 weeks postinjury (n = 6). b,c) LSS scores Photographs of swimming test at 56 days postinjury (n = 6). d, e) Representative images of footprint tracks and gait patterns. Left forelimb (yellow), LF; right forelimb (blue), RF; left hindlimb (green), LH; right hindlimb (purple), RH. f-k) Statistical analysis of six commonly used catwalk parameters (regularity index, max contact area, duty cycle, step cycle, stride length, and print position) (n = 6). (ns indicates no significant, ∗p < 0.05, ∗∗p < 0.01, ∗∗∗p < 0.001, ∗∗∗∗p < 0.0001). (For interpretation of the references to colour in this figure legend, the reader is referred to the Web version of this article.)

Based on our preliminary findings, we conducted further analysis to explore the effects of these therapeutic interventions on the recovery of locomotor function in a rat model of SCI. We calculated the BBB scores at weeks 1, 2, 3, 4, 5, 6, 7, and 8, which indicated that the hindlimb function of rats with SCI was severely impaired and resulted in paralysis. Moreover, all experimental groups exhibited varying degrees of recovery in locomotor function, with statistically significant differences observed between groups at 7 and 8 weeks. Encouragingly, the results demonstrated that the WPC hydrogel with wireless ES effectively facilitated the recovery of locomotor function of rats. In contrast, the other single-treatment groups showed no such effect (Fig. 10a). In the swimming test, rats that received the WPC-US treatment showed a reduced angle between their trunk and the water surface, along with an increased frequency of hindlimb movements, indicative of improved motor function. The WPC-US group achieved higher LSS scores than other groups (Fig. 10b and c).

The CatWalk XT system is an advanced, automated platform for gait analysis, which uses high-speed cameras to capture footprints and derive gait parameters, thus evaluating the locomotive capabilities of rats. According to the CatWalk footprint analysis, the hindlimb motor function of rats in the SCI group was markedly compromised, resulting in disordered gait and reduced support strength during ambulation whereas clear footprints and coordinated gait were visible in the treatment groups, especially in WPC-US group (Fig. 10d and e). Analysis of the six commonly used catwalk parameters (regularity index, max contact area, duty cycle, step cycle, stride length, and print position) showed that the WPC hydrogel with WES could significantly improve the recovery of locomotor function (Fig. 10f–k).

### 3.9 WPC hydrogel with WES improves neurogenesis and axonal regeneration in vivo

**Fig. 11:**
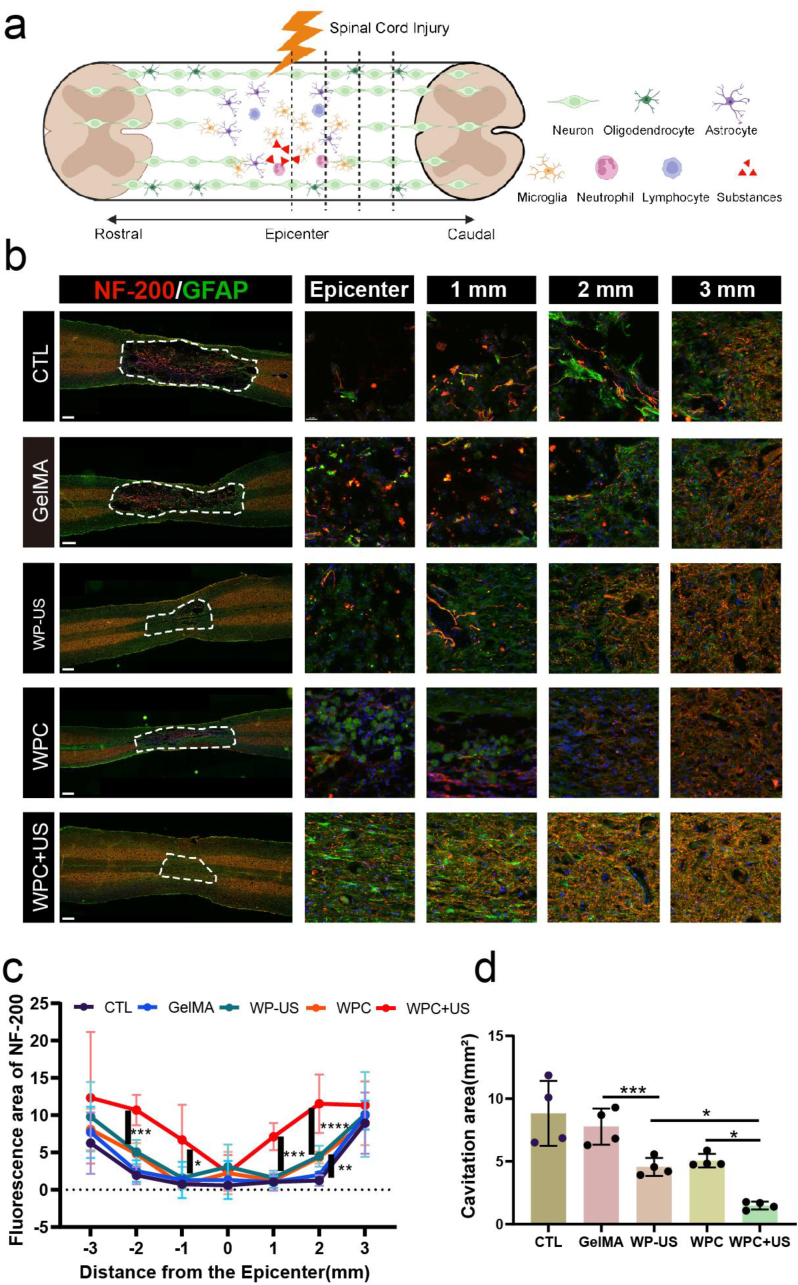
The WPC hydrogel with wireless ES facilitates axonal regeneration and remodeling of the neuronal network after SCI. a) Schematic of the measured area of spinal cord gray matter. (Created with BioRender.com.) b) Immunofluorescence staining of the injured area 28 days after SCI. Red represents NF200-positive, and green represents GFAP-positive. Scale bars: 500 μm. Scale bars of magnified image: 30 μm. c) Quantification o the fluorescence intensity at different distances to the epicenter. d) Cavitation area of lesion core. (∗p < 0.05, ∗∗p < 0.01, ∗∗∗p < 0.001, ∗∗∗∗p < 0.0001). (For interpretation of the references to colour in this figure legend, the reader is referred to the Web version of this article.)

Axonal outgrowth and synapse formation are vital to rebuild neural circuits for functional recovery, which we further evaluated at 8 weeks after SCI. We applied the biomarkers GFAP and NF200 to label astrocytes and neurofilaments, respectively. As depicted in Fig. 11 a, we evaluated the fluorescence area of NF200+ staining at 1, 2, and 3 mm, both caudal and rostral, from the lesion epicenter. As shown in Fig. 11b and c, WPC-US treatment resulted in a significant sparing of neurons spanning 2 mm caudal from the epicenter compared with that in the WP-US and WPC groups. Immunofluorescence staining revealed substantial cavitation in the SCI and GelMA groups at 8-week. However, an obvious reduction in cavitation was observed in both the WP-US and WPC groups. In particular, the WPC-US group exhibited minimal cavitation area among the treatment groups (Fig. 11d). These data showed that wireless ES driven by US of WPC hydrogel promoted axonal regeneration and cavitation reduction.

## 4 Discussion

SCI inevitably results in severe functional impairment, posing significant challenges for recovery. Accumulated evidence suggests that microenvironment imbalances and limited intrinsic regeneration are pivotal obstacles that prevent recovery [34]. Stem cell therapies have been demonstrated promising for SCI because of serving as a source of neural cells. Nevertheless, severe inflammation, microenvironment imbalances, and other pathological conditions lead to poor neural regeneration outcomes. Increasing evidence demonstrates that cotransplantation of different stem cells represents a potential strategy toward effective regeneration. For instance, Hu et al. reported that Schwann cells (SCs) could promote the survival, proliferation, and migration of glial-restricted precursor cells (GRPs), and cografts of GRPs and SCs promoted the recovery of function after SCI [35]. Goni et al. also demonstrated the safety and feasibility of cotransplanting bone marrow-derived MSCs and olfactory mucosa in patients with thoracic-level SCIs [36].

Cell therapy is a promising strategy for SCI treatment. NSCs are considered the optimal cellular candidates for restoring the central nervous system due to their multifaceted therapeutic potential. NSCs possess the remarkable capacity to differentiate into various mature cell types, with particular significance in their ability to differentiate into functional neurons and glial cells, which are required for the proper functioning of the nervous system. NSCs could recover myelin sheaths, reconstitute neuronal circuitry, provide trophic support, and replace damaged neural tissue by bridging lesions, which is crucial for the reestablishment of functional neural connections. Extensive research has confirmed that transplantation of NSCs results in significant functional recovery after SCI [37,38]. However, their application is limited by microenvironment imbalances in SCI, which results in massive cell death [39]. To summarize, the poor survival rate, limited proliferation capacity, and suboptimal differentiation potential of NSCs have emerged as major obstacles in SCI treatment [40]. hUCMSCs, distinguished by their rapid proliferation, phenotypic plasticity, and multilineage differentiation capabilities, are recognized as a valuable resource in the field of regenerative medicine and are also considered a promising therapeutic method for SCI. hUCMSCs exhibit remarkable autocrine and paracrine activities that regulate immune system components and microenviroment [41]. hUCMSCs can provide a comprehensive microenvironment enriched with various growth factors, nutrients, and regulatory molecules. This microenvironment plays a supportive role for NSCs through the paracrine signaling and immunomodulatory effects of hUCMSCs.

In this study, we established a coculture system of hUCMSCs and NSCs and observed that hUCMSCs could promote the proliferation and differentiation of NSCs. Quantitative analysis confirmed that hUCMSCs can facilitate the differentiation of NSCs toward neurons. Furthermore, Sholl analysis revealed that hUCMSCs could improve the growth of axons in differentiated neurons and improve synaptic plasticity. To further explore the underlying mechanisms, we conducted mRNA transcriptome sequencing on NSCs. RNA-sequencing analysis and western blot validation confirmed that hUCMSCs regulate the directed differentiation of NSCs through the PDK1-driven PI3K/AKT signaling pathway.

In addition to remodeling tissue structure, it is imperative that we focus on bioelectrical signal recovery. The profound impact of bioelectrical signals on neural regeneration in SCIs has rendered the development of spinal cord stimulation a topic of considerable interest in recent years [42]. Nevertheless, the spinal cord stimulators currently used in the clinic require invasive electrodes, bulk batteries, implantable devices. Therefore, piezoelectric nanogenerators provide a novel solution to this problem. Accordingly, we designed a WP hydrogel fabricated with PDA@BT NPs and conductive polymer PEDOT that could generate electrical signals under US.

Neurons are the fundamental units of the nervous system, and recent research has emphasized the importance of electrical signals in neurogenesis and the morphogenesis of axons and dendrites. To investigate the effect of WES, we performed in vitro stimulation of neurons with the WP hydrogel. Our results showed that WES facilitated and improved neurite outgrowth in neurons. We also observed that WES activated the Piezo1 channel, which resulted in Ca^2+^ influx in primary cortical neurons. The calcium signaling pathway is a crucial component of intracellular communication that regulates a diverse array of cellular processes. This pathway is initiated by the influx of Ca^2+^ into the cytoplasm, which can be triggered by various stimuli. Calcium influx within the nervous system plays a vital role in synaptic plasticity and is crucial for axonal growth and neural development [43]. Moreover, WES significantly increased the phosphorylation of CaMKII and CREB in neurons. Both these proteins are important players in neuronal Ca^2+^ signaling. CaMKII is directly regulated by calmodulin, a good indicator of Ca^2+^ level inside the cell, and both CaMKII and CREB are involved in critical neuronal functions such as neurotransmitter secretion, plasticity, transcription regulation, learning, and memory [44]. However, when neurons were treated with both WES and GsMTx4, an inhibitor of Piezo1, there was no activation of CaMKII and CREB, suggesting that WES exerts its neuromodulatory effects by targeting the Piezo1/Ca^2+^/CaMKII/CREB pathway, indicating the importance of this pathway in mediating the effects of WES on neuronal function.

Compared with direct transplantation, transplanting cells through tissue engineering can better simulate the ECM, provide a stable microenvironment, and further promote nerve regeneration and functional recovery. The application of PDA@BT in neuroregeneration is hindered by chemical/mechanical incompatibility interfaces [17]. GelMA exhibits excellent biocompatibility and mimics the mechanical properties of neural tissue, making it suitable for filling injury cavities and promoting neural tissue regeneration. Nevertheless, its limited conductivity constrains its application in modulating the excitability of neural cells. Good biosecurity is the basic requirement for biomaterial applications. Studies have confirmed that the GelMA/PEDOT:PSS conductive hydrogel PEDOT:PSS improves dorsal root ganglion and protects neurons [22,30]. The selected concentration of PEDOT:PSS of 1 mg/mL was based on the literature [31,32]. In this study, we incorporated PDA@BT NPs into GelMA/PEDOT:PSS to construct the WP hydrogel. Then we fabricated the WPC hydrogel by incorporating NSCs and hUCMSCs as seed cells into the WP hydrogel. To investigate the effects of WES through the WPC hydrogel in vivo, we generated a rat model with a T10 segmental SCI, resulting in an immediate significant hematoma at the injury site. SCI initiates a cascade of detrimental effects. Typically, within 1 week postinjury, a cystic cavity begins to form and gradually enlarges during subacute phase, which is often accompanied by progressive secondary damage, encompassing inflammation, oxidative stress, and apoptosis [45]. Therefore, we administered WPC hydrogels directly into the cystic cavities 1 week postinjury to evaluate their therapeutic potential. By injecting the WPC hydrogel, the hydrogel can be filled into the cystic cavities of clinically relevant contusion injury and generate microelectrical signals driven by US on the injured tissues. Meanwhile, the US intensity applied to the spinal cord in this study (2 W/cm^2^) is comparable to that of current clinical treatments, indicating a relatively low potential risk.

In the subacute phase of SCI, microglia activate and release inflammatory mediators. This initial response serves to recruit and activate macrophages, thereby exacerbating the imbalance in the microenvironment. Macrophages possess the plasticity to exert either beneficial or detrimental effects on tissue outcomes, depending on their activated phenotype. The M1/M2 macrophage classification system, which delineates these functional dichotomies, was first articulated in a foundational study by Mills et al. [46] After an injury, macrophages initially adopt a proinflammatory phenotype (M1) to secrete inflammatory cytokines. Macrophages with a pro-regenerative phenotype, known as M2 macrophages, are essential for resolving inflammation in a time-dependent manner. They facilitate this process by secreting signaling molecules, including anti-inflammatory cytokines and growth factors, and by phagocytosing cellular debris, which promotes tissue remodeling [47]. The transition from M1 to M2 and the maintenance of a balance between the two phenotypes is crucial for the healing process after SCI.

Scar stiffness is closely associated with the formation of cystic cavities in chronic phase. The persistent presence of scar tissue poses a physical barrier to axonal regeneration and is widely recognized as a major obstacle in recovering nerve function, making it a critical target in SCI treatment. Matrix stiffness is a critical mechanical property that is altered after SCI due to the formation of scar tissue and excessive collagen fiber deposition. Scar tissue formation after SCI is accompanied by collagen fiber deposition, which increases the stiffness of the scar matrix compared with the surrounding normal neural tissue. High stiffness in scar tissue acts as a mechanical barrier, significantly preventing axonal penetration through the scar matrix. Cooper et al. demonstrated that SCI induces chronic mechanical stiffening in a mouse model and contributes to the limited regenerative capacity of the damaged spinal cord [48]. In addition, reducing scar tissue stiffness has been demonstrated to improve repair outcomes in SCI by generating a more permissive microenvironment for tissue regeneration.

While scar tissue stiffness significantly hampers axonal regeneration, it also plays a critical role in the formation of cystic cavities, which may exert a profoundly detrimental effect in SCI. More than half of the patients may develop posttraumatic spinal cord cysts, or syringomyelia, which typically manifests at the epicenter of the injury. The cystic cavity that forms after injuries in the spinal cord presents a significant impediment to tissue repair within the central nervous system. Cavity space hinders the infiltration of cellular elements essential for axonal regeneration, posing a significant barrier to the healing process. Our results indicated that the synergistic therapeutic effectively reduced scar stiffness and mitigated the formation of cystic cavities by softening the ECM stiffness and alleviating mechanical barriers. Hence, our strategy helps in reconstructing scar-associated cavities and improves the integration of regenerating tissues within the injury site. These findings emphasize the importance of targeting mechanical properties, such as matrix stiffness, as a potential therapeutic approach to promote spinal cord repair and functional recovery.

To summarize, we demonstrated that the WPC hydrogel induces a direct phenotypic switch from M1 to M2 polarization of macrophages, regulating the immune microenvironment. In the chronic stage, the WPC hydrogel could reduce the volume of cystic cavities, exert bridging effects on posttraumatic cavities, and support axonal growth. The WPC hydrogel, when combined with WES, could act complementarily and synergistically to further promote greater anatomical and functional restoration.

Nonetheless, there are several limitations and challenges that could be further investigated in future research. First, to better reflect the therapeutic effects of SCI, transection injury models should be considered. Second, the distribution of the hydrogel and the parameters of in vivo ES after injection require investigation. Finally, we intend to focus on other immune cell types to further clarify the underlying mechanisms.

## 5 Conclusion

We developed a WPC hydrogel, which could serve in situ WES and synergize with the cotransplantation of NSCs–hUCMSCs to promote recovery after SCI. We constructed a coculture system of NSCs and hUCMSCs and found that hUCMSCs could promote NSC proliferation and differentiation through the PDK1-driven PI3K/AKT signaling pathway. When a safe US was used in a WP hydrogel, the in situ wireless ES could trigger a significant increase in the spontaneous electrical activity of neurons in vitro. Through Sholl analysis, we discovered that wireless ES can facilitate the dendritic complexity of neurons through Piezo1 channel activation and downstream CaMKII/CREB signaling. In rats with spinal cord contusion injury, there was a significant improvement in locomotor and neural function in the WPC-US group. Immunofluorescence and imaging data suggested that the WPC hydrogel with in situ wireless ES could effectively reduce scar stiffness, restructure scar cavities, and accelerate axonal regeneration. Therefore, our strategy of wireless in situ ES synergizing with the cotransplantation of NSCs–hUCMSCs represents a novel path to improve spinal cord regeneration in neural tissue engineering.

## CRediT authorship contribution statement

**Hao Zhong:** Writing – original draft, Visualization, Validation, Formal analysis. **Mi Zhou:** Writing – original draft, Visualization, Validation, Methodology, Formal analysis. **Junrui Guo:** Visualization, Validation, Software, Formal analysis. **Danyang Chen:** Validation, Methodology, Formal analysis. **Cong Xing:** Visualization, Validation, Methodology. **Song Liu:** Validation, Methodology. **Hongjiang Yang:** Validation, Methodology, Formal analysis. **Hongpeng Ma:** Visualization, Validation. **Qi Zhang:** Software, Methodology. **Jianhai Yang:** Writing – review & editing, Conceptualization. **Shiqing Feng:** Writing – review & editing, Supervision, Project administration. **Guangzhi Ning:** Writing – review & editing, Project administration, Conceptualization.

## Ethics approval and consent to participate

All animal experiments were approved by the Animal Ethics Committee at Tianjin Medical University General Hospital.

## Consent for publication

All the authors read and approved the manuscript.

## Declaration of competing interest

The authors declare that they have no known competing financial interests or personal relationships that could have appeared to influence the work reported in this paper.

## Data Availability

Data will be made available on request.
