# Supplementary material for: Ultrasound-driven wireless piezoelectric hydrogel synergizes with cotransplantation of NSCs–hUCMSCs for structural and functional recovery in spinal cord injury
Source: Mater Today Bio. 2025 Apr 26;32:101805. doi: 10.1016/j.mtbio.2025.101805 (PMC12088769; doi:10.1016/j.mtbio.2025.101805)
Supplement: Multimedia component 1 [file mmc1.docx]

**Supporting Information**


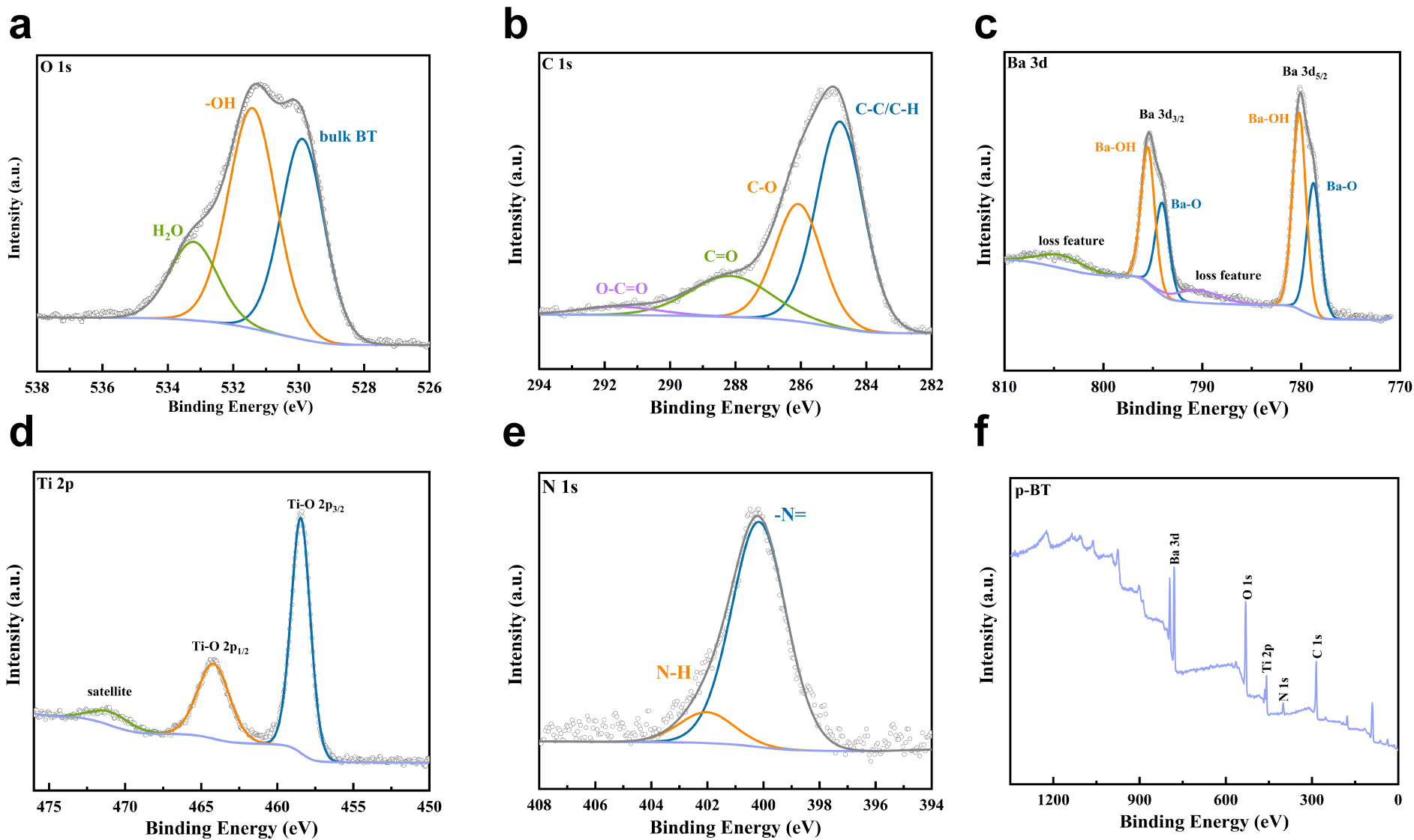


Figure S1. The XPS survey spectrum of PDA@BT. a) o1s, b) O 1s, c) Ba 3d, d)Ti 2p, e) N 1s and surey f) XPS spectrums of the PDA@BT.


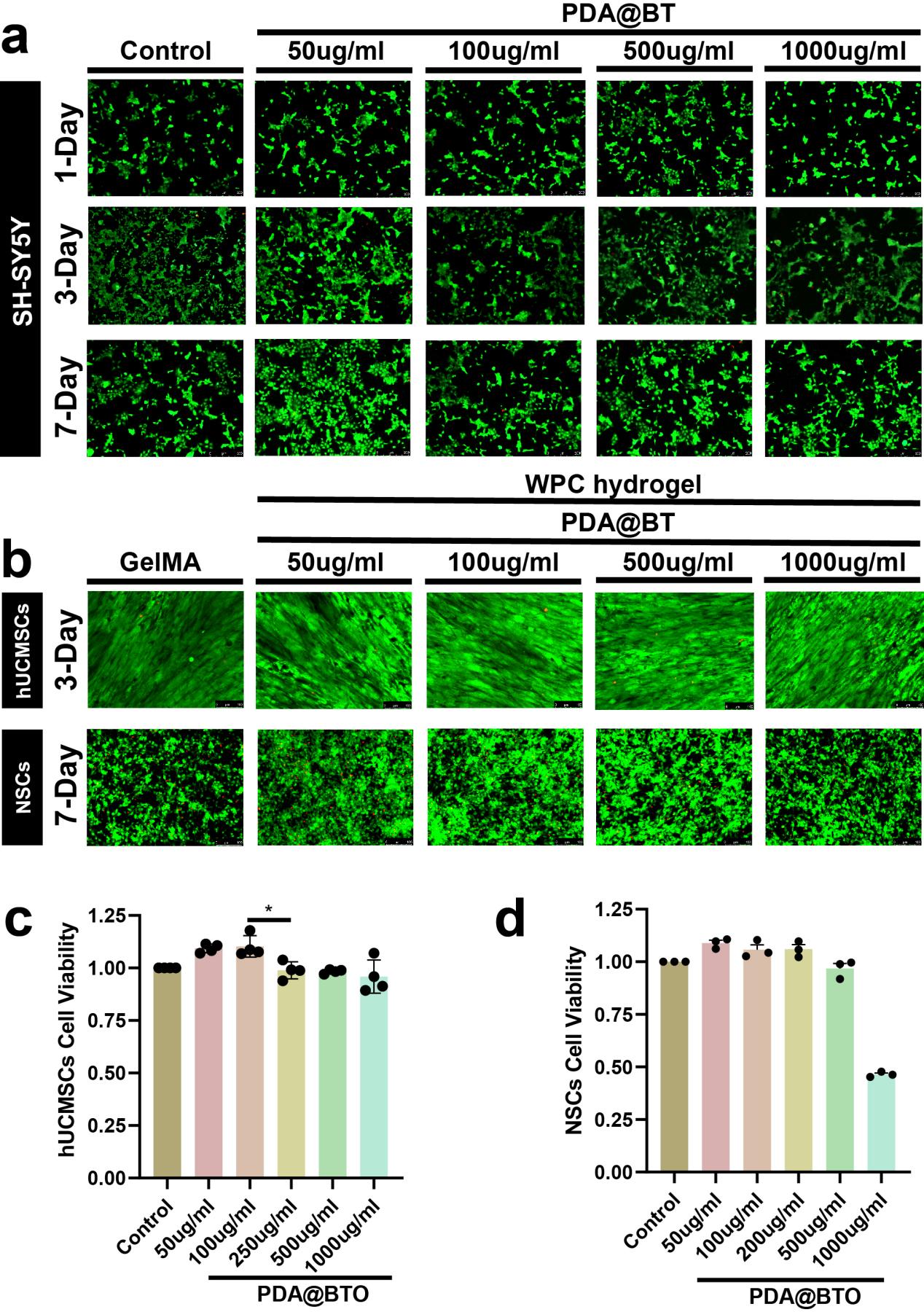


Figure S2. Cells compatibility. a)Live-dead cell staining of SH-SY5Y cells cultured with hydrogel leachate for 24 hours at 1, 3, and 7 days. b) Live-dead cell staining of hUCMSCs and NSCs cultured for 7 days. c, d) Cell counting kit 8 (CCK-8) test was used to evaluate the cytotoxicity and optimal concentration of PDA@BT NPs on hUCMSCs and NSCs.


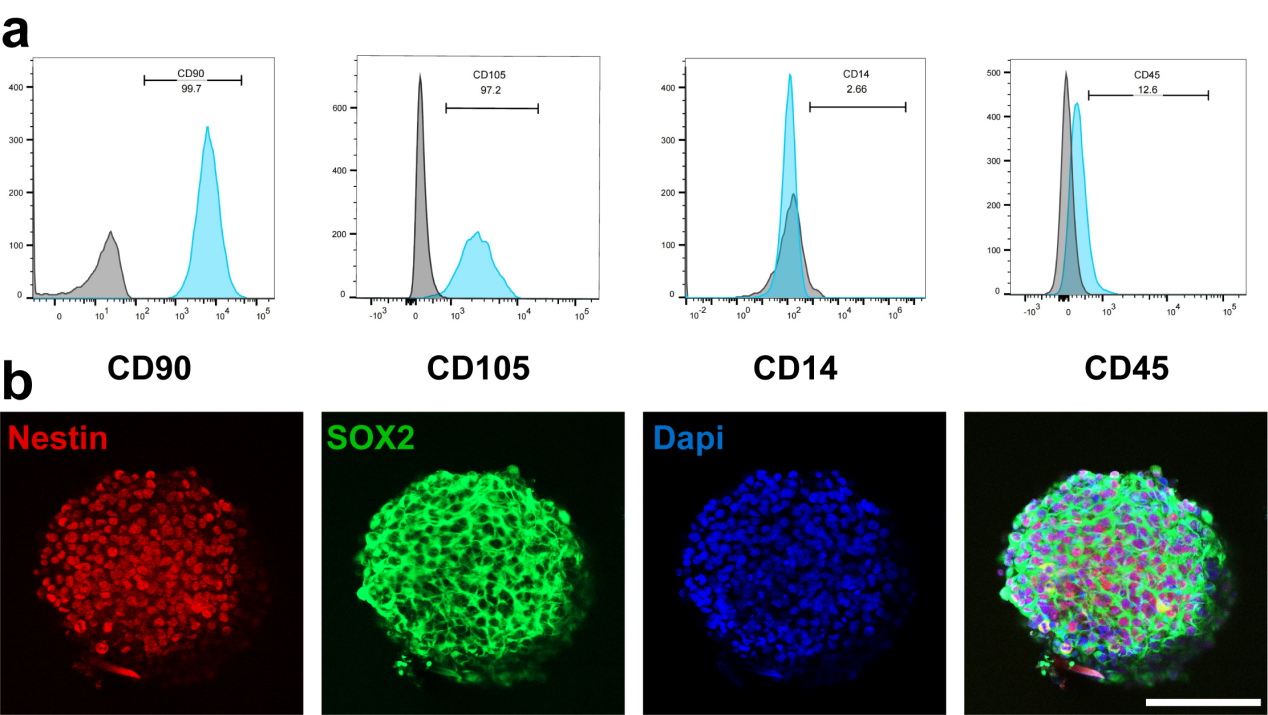


Figure S3. Cells identification. a) Flow-cytometric analysis of HUCMSCs. b) Immunofluorescence analysis of NSCs. Scale bars = 100 µm.


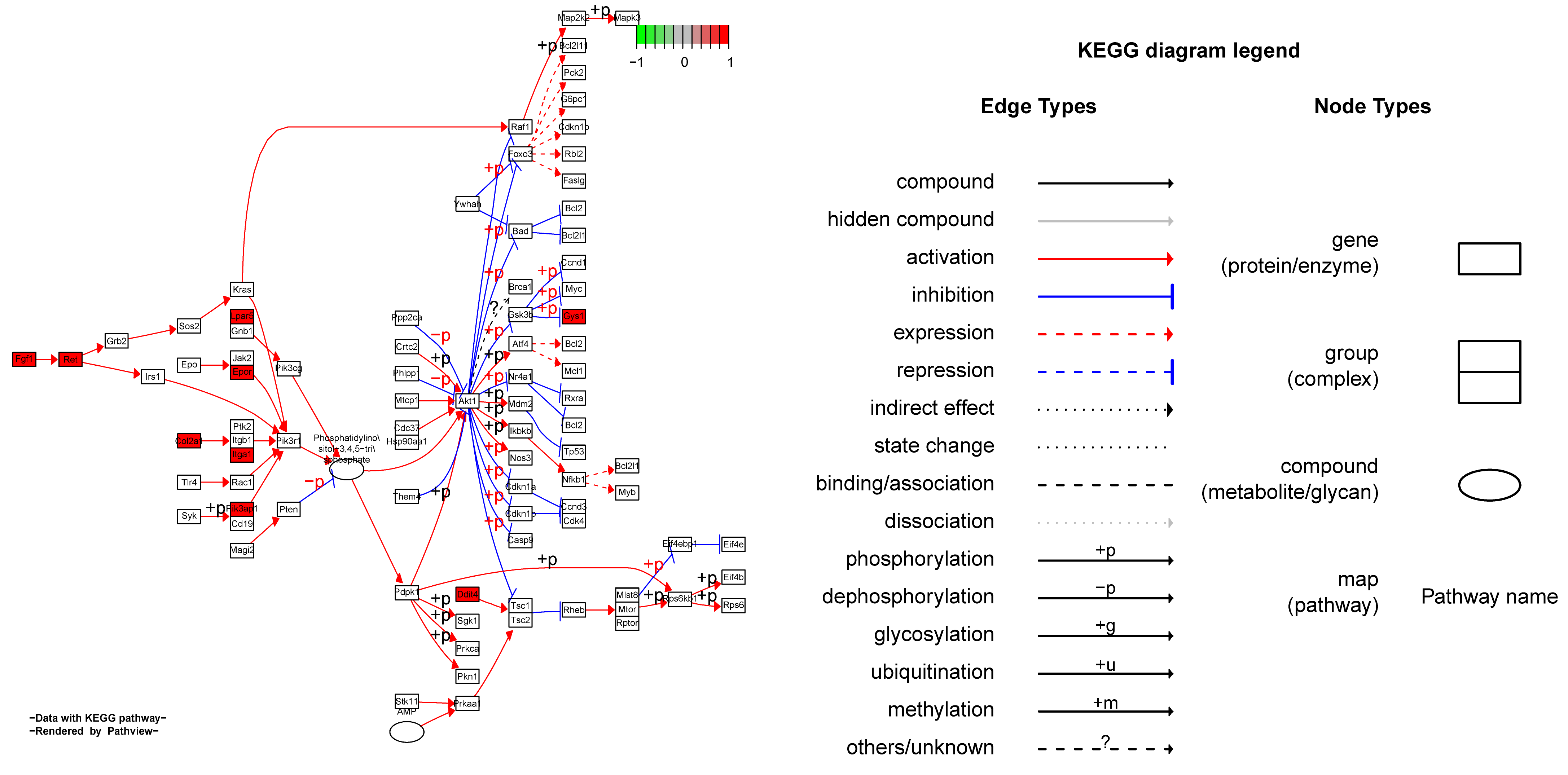


Figure S4. Enrichment analysis of genes related to the PI3K-AKT pathway.

**Supporting Table 1**

| **ONTOLOGY** | **ID** | **Description** |
| --- | --- | --- |
| BP | GO:0030595 | leukocyte chemotaxis |
| BP | GO:0006935 | chemotaxis |
| BP | GO:0042330 | taxis |
| BP | GO:0060326 | cell chemotaxis |
| BP | GO:0070661 | leukocyte proliferation |
| BP | GO:0050900 | leukocyte migration |
| CC | GO:0031012 | extracellular matrix |
| CC | GO:0030312 | external encapsulating structure |
| CC | GO:0062023 | collagen-containing extracellular matrix |
| CC | GO:0043235 | receptor complex |
| CC | GO:0009897 | external side of plasma membrane |
| CC | GO:0098802 | plasma membrane signaling receptor complex |
| MF | GO:0005539 | glycosaminoglycan binding |
| MF | GO:0030246 | carbohydrate binding |
| MF | GO:0030546 | signaling receptor activator activity |
| MF | GO:0048018 | receptor ligand activity |
| MF | GO:0008201 | heparin binding |
| MF | GO:0048029 | monosaccharide binding |
